# Crystal Structure, Topology, DFT and Hirshfeld Surface Analysis of a Novel Charge Transfer Complex (L3) of Anthraquinone and 4-{[(anthracen-9-yl)meth-yl] amino}-benzoic Acid (L2) Exhibiting Photocatalytic Properties: An Experimental and Theoretical Approach

**DOI:** 10.3390/molecules27051724

**Published:** 2022-03-06

**Authors:** Adeeba Ahmed, Aysha Fatima, Sonam Shakya, Qazi Inamur Rahman, Musheer Ahmad, Saleem Javed, Huda Salem AlSalem, Aiman Ahmad

**Affiliations:** 1 Department of Applied Chemistry, Zakir Husain College of Engineering and Technology, Faculty of Engineering and Technology, Aligarh Muslim University, Aligarh 202002, UP, India; adeebaamu10@gmail.com (A.A.); amusheer4@gmail.com (M.A.); 2 School of Studies in Chemistry, Jiwaji University, Gwalior 474011, MP, India; fatimaaisha27@gmail.com; 3 Narayan Laxman Mahavidyalaya, Basant Nagar, Gaisinghpur, Farrukhabad 209602, UP, India; sonamshakya08@gmail.com; 4 Department of Chemistry, Integral University, Lucknow 226026, UP, India; qirahman@iul.ac.in; 5 Department of Chemistry, Institute of H. Science, Khandari, Dr. Bhimrao Ambedkar University, Agra 282002, UP, India; saleem.7javed@gmail.com; 6 Department of Chemistry, College of Science, Princess Nourah bint Abdulrahman University, P.O. Box 84428, Riyadh 11671, Saudi Arabia

**Keywords:** donor–acceptor, crystal studies, hirshfeld surface analysis, DFT, photocatalysis, molecular docking

## Abstract

Here, we report a facile route to the synthesizing of a new donor–acceptor complex, L3, using 4-{[(anthracen-9-yl)meth-yl] amino}-benzoic acid, L2, as donor moiety with anthraquinone as an acceptor moiety. The formation of donor–acceptor complex L3 was facilitated via H-bonding and characterized by single-crystal X-ray diffraction. The X-ray diffraction results confirmed the synthesized donor–acceptor complex L3 crystal belongs to the triclinic system possessing the P-1 space group. The complex L3 was also characterized by other spectral techniques, viz., FTIR and UV absorption spectroscopy, which confirmed the formation of new bonds between donor L2 moiety and acceptor anthraquinone molecule. The crystallinity and thermal stability of the newly synthesized complex L3 was confirmed by powdered XRD and TGA analysis and theoretical studies; Hirshfeld surface analysis was performed to define the type of interactions occurring in the complex L3. Interestingly, theoretical results were successfully corroborated with experimental results of FTIR and UV absorption. The density functional theory (DFT) calculations were employed for HOMO to LUMO; the energy gap (∆E) was calculated to be 3.6463 eV. The complex L3 was employed as a photocatalyst for the degradation of MB dye and was found to be quite efficient. The results showed MB dye degraded about 90% in 200 min and followed the pseudo-first-order kinetic with rate constant k = 0.0111 min^−1^ and R^2^ = 0.9596. Additionally, molecular docking reveals that the lowest binding energy was −10.8 Kcal/mol which indicates that the L3 complex may be further studied for its biological applications.

## 1. Introduction

In recent years, several attempts have been made regarding the synthesis of charge transfer (CT) complexes due to their structural applications in different branches of chemistry including organic semiconductors [1,2], photocatalysts [3], biological complexes [4,5,6], liquid crystals [7] and many more. Owing to their importance, since they can also act as an intermediate molecule or can have transitory existence in some important chemical reactions, efforts have been made by scientists to understand their synthetic applications. The most important aspect of CT complexes is their intermolecular interactions in the field of coordination and supramolecular chemistry. The existence of hydrogen bonding and non-covalent interactions make them obtain more attention in the emerging field of supramolecular chemistry [8,9]. Donor–acceptor molecular systems are also parts of these kinds of systems, and in this respect, we have synthesized a new donor–acceptor system (L3) by synthesizing our own ligand (L2) [10] as donor and anthraquinone as an acceptor molecule. Anthraquinone has been an important chemical for pharmaceutical preparations [11]. The reason anthraquinone is biologically active is the presence of the quinone system [12,13]. In most of the cases of CT complexes with an anthraquinone- or quinone-based system, the quinone moiety acts as an acceptor of electrons; this is the reason that they are in demand in redox chemistry [14,15]. Experimental data were justified with DFT/TD-DFT B3LYP/6-311++G(d,p) (basis set) theoretical calculations to obtain chemical, structural, spectroscopic, thermodynamic and vibrational phenomena of the complex L3. Until now, the CT complexes of anthraquinone had been less explored. We have successfully synthesized a novel CT complex of anthraquinone with our own synthesized ligand L2.

Methylene blue (MB) is a basic dye that is widely utilized in different industries, viz., coloring paper, dyeing cotton and wools, leather, etc. [16]. Direct discharge of these industrial effluents which contain MB dye can cause serious damaged to the environment as well as to human health [17]. Due to its slow rate of degradation, it persists in the environment for long period of time. Therefore, it is of utmost importance to treat these industrial effluents well before they are discharged into water. Physical or chemical methods are commonly employed for effective removal of dye-degradation products, viz., adsorption, biodegradation, reverse osmosis, coagulation, flocculation, ion exchange, advanced oxidation process and heterogeneous photocatalysis [18,19]. Among them, heterogeneous photocatalysis is commonly employed to eliminate these hazardous effluents by turning them into less-toxic materials such as carbon dioxide, water and other less-harmful materials [20,21,22]. In recent years, CT complexes are also being explored for their photocatalytic property in dye degradation [23]. Here, we have reported the successful facile degradation of MB dye from artificially contaminated water through the photocatalytic degradation process. The CT complexes in this paper were studied theoretically via the B3LYP/6–311++G(d,p) basis set. For the vibrational frequency analysis (FT-IR) and UV–Visible spectra analysis, experimental and theoretical studies were carried out. To determine the reactive sites in the molecule and its inclusive reactivity, molecular electrostatic potential (MEP) surface simulation was performed. Intermolecular interactions were investigated in 3D and 2D surfaces in the crystal structure of the molecule using Hirshfeld surface analysis. The molecular docking was also carried out with a protein of the oxidoreductase domain to assess for drug potentiality and efficacy. The density of states (DOS) and partial density of states (p-DOS) calculations were completed with the help of band-gap calculations to determine the probable mechanistic pathway [24].

## 2. Material and Methods

### 2.1. Reagents and Materials

All the materials used were of high grade without further purification. 4-amino benzoic acid, 9-anthracenecarboxaldehyde and anthraquinone were purchased from Sigma Aldrich (St. Louis, MO, USA) and used as such. The solvent used was ethanol (analytical grade) used as such and purchased from Fisher Scientific, Bangalore, India.

### 2.2. Methods and Instrumentation

The IR spectra of the synthesized complex were measured on Thermo Scientific iS50-FTIR spectrophotometer (India Pvt. Ltd., Bangalore, India) in the range from 4000 to 400 cm^−1^ by using KBr pellets. A Thermo Scientific Evolution 201 UV–Vis spectrophotometer (Waltham, MA, USA) was used for the absorption spectrophotometric measurements of both L2 and L3. The samples were prepared in 10^−4^ M concentration in a cuvette of 1 cm path length with ethanol as solvent. The PXRD pattern of the CT complex (L3) was recorded using the Miniflexll X-ray Diffractometer (sourced from I R Technology Services Pvt. Ltd., Maharashtra, India) with Cu-Kα radiation scan in the range of 4 to 80 (as 2theta value). The Thermogravimetric analysis (TGA) curve was recorded on a Shimadzu TGA-50H instrument (Kyoto, Japan) under nitrogen atmosphere at a heating rate of 20 °C min^−1^.

### 2.3. Synthesis of CT Complex (L3)

**Scheme 1:**
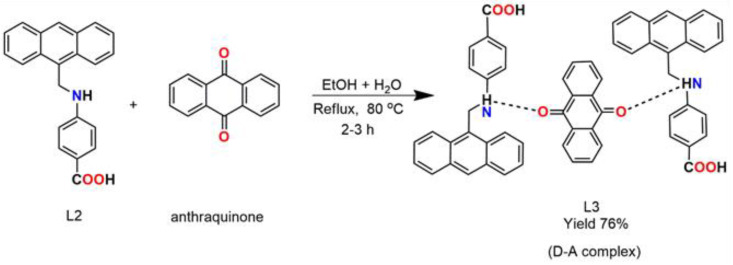
Synthetic scheme for donor–acceptor, D–A, complex, (L3).

The ligand (L2) was synthesized by an earlier reported procedure [10]. Both ligand (L2) and anthraquinone were taken in 1:1 ratio; L2 (0.459 mmol, 0.015 g) and anthraquinone (0.0459 mmol, 0.0095 g) were placed in a 100 mL round-bottom flask and dissolved in a solvent of ethanol and water in a ratio of 1:0.5, and the mixture was refluxed at 80 °C for 2 h. A clear yellow-colored solution obtained was allowed to cool and kept for slow evaporation after filtration (Scheme 1). After 10 to 15 days, yellowish-brown-colored crystals were obtained, which were characterized by SCXRD. Yield: 76%. Elemental analysis was performed with the CE-440 Elemental Analyzer (Exeter Analytical, Inc. North Chelmsford MA, USA) and the calculated elemental analysis (%) for C_58_H_41_N_2_O_6_ (861.98) was C, 80.82; H, 4.79; N, 3.25%; the found percentages were C, 79.92; H, 4.25; N, 3.09%.

### 2.4. Photodegradation of Dye

To determine the photocatalytic activity of the as-synthesized complex (L3), we successfully monitored dye degradation for MB, a cationic dye, at ambient temperature. The photocatalytic experiment was performed in the Pyrex flask reactor under light illumination using a xenon arc lamp. For an efficient dye-degradation reaction, an appropriate amount (100 mg) of synthesized complex (L3) was added into a 250 mL MB dye solution of 20 ppm strength. Before the illumination of light, the aqueous suspension of MB dye with the photocatalyst (complex L3) was set to continuous stirring for 60 min to develop adsorption–desorption equilibrium in the dark. Afterwards, the suspension of stable dye was illuminated by UV light. Then, we measured the decomposed dye’s absorption behavior periodically every 20 min by a UV–Visible spectrophotometer (PerkinElmer Model 750, Shelton, CT, USA). With the equation

ln (C_0_/C_t_) = *k*t

where C_0_ is the initial concentration of dye at time t = 0, while C_t_ represents dye concentration at time t = t, a plot between ln(C_0_/C_t_) vs. time intervals gives a straight line with slope *k* [25]. The control experiment was performed under the same conditions but in the absence of the synthesized complex (L3) catalyst.

## 3. Results and Discussion

### 3.1. Structure Description

**Figure 1:**
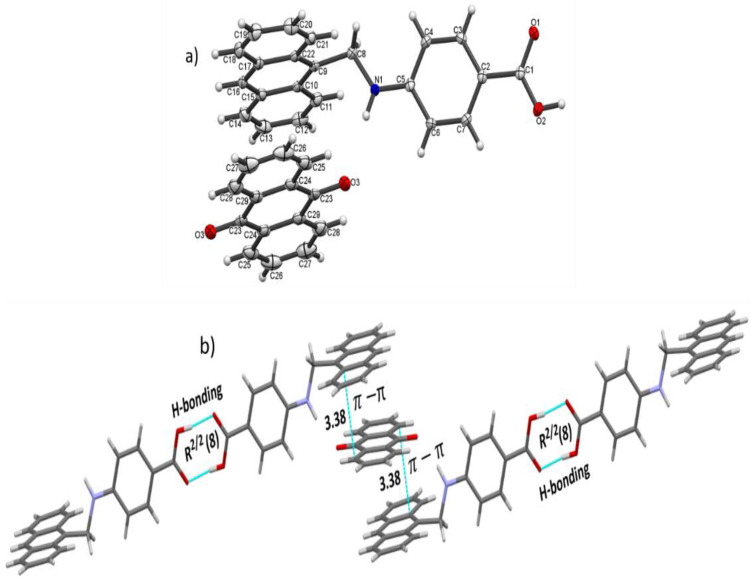
(**a**) Asymmetric unit of complex L3 in ORTEP view; (**b**) extended network representing π–π stacking and H-bonding in the complex L3.

**Figure 2:**
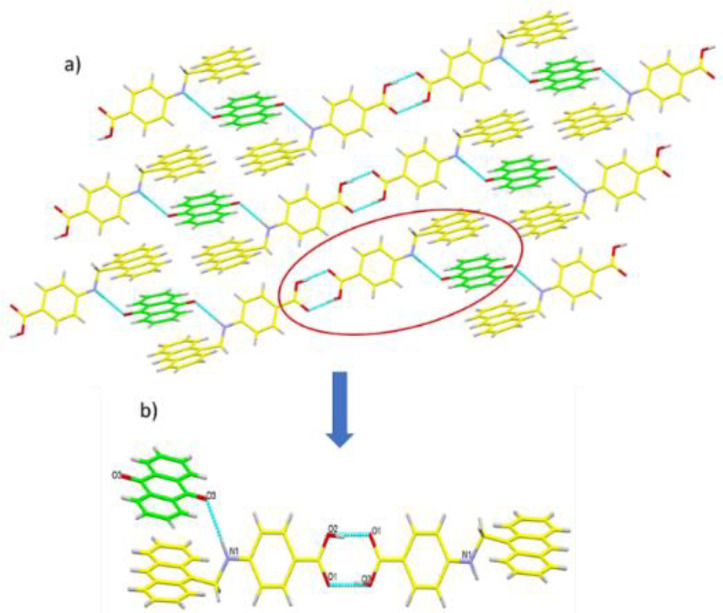
(**a**) Extended H-bonding network between L2 and anthraquinone; (**b**) inter and intramolecular H-bonding between N1–O3 and O2–O1, respectively.

Single-crystal X-ray structure analysis revealed that L3 crystallizes in the triclinic system with the P-1 space group (Table 1). The ORTEP view of the complex illustrates the coexistence of both anthraquinone and L2 ligand in the D–A complex (Figure 1a). The crystal structure also exposes that there is inter as well as intramolecular H-bonding between N1–O3 and O2–O1 of one L2 molecule to another L2 molecule (Figure 2). The interatomic distance between N1–O3 was found to be 2.98 Å; furthermore, the dihedral between the two planes of the L2 moiety and the anthraquinone moiety was 14.064(12). The torsional angle between the C_aryl_-CH_2_-NH-C_aryl_ of the L2 moiety was found to be 175.9(2°). The extension of the asymmetric unit via short contacts have elaborated the π–π stacking between the rings of anthraquinone and ligand (L2) along with H-bonding between the carboxylic groups of moieties to the other (Figure 1b), which confirms the interaction between the acceptor and donor molecule. The bond lengths, bond angles and other parameters are provided in Supplementary Materials, Tables S1–S5.

### 3.2. Topological Analysis

**Figure 3:**
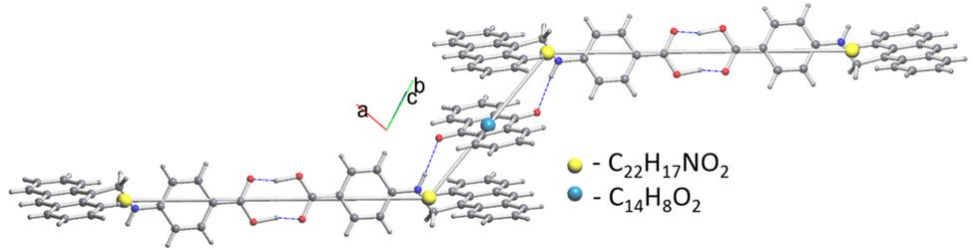
2C1 topology of 2-c net in standard representation of H-bonded molecular structure of L3, corresponding to the nodes of the underlying net.

Topological analysis was also performed for L3 to examine the weak interaction existing in the molecular framework, which resulted in a 1D periodic structure due to H-bonds (Supplementary Materials, Figure S1). The H-bonded structure, when represented in standard form, exposed the 2-c uninodal net of the 2C1 topological type with a point symbol for the net: {0} (Figure 3).

**Figure 4:**
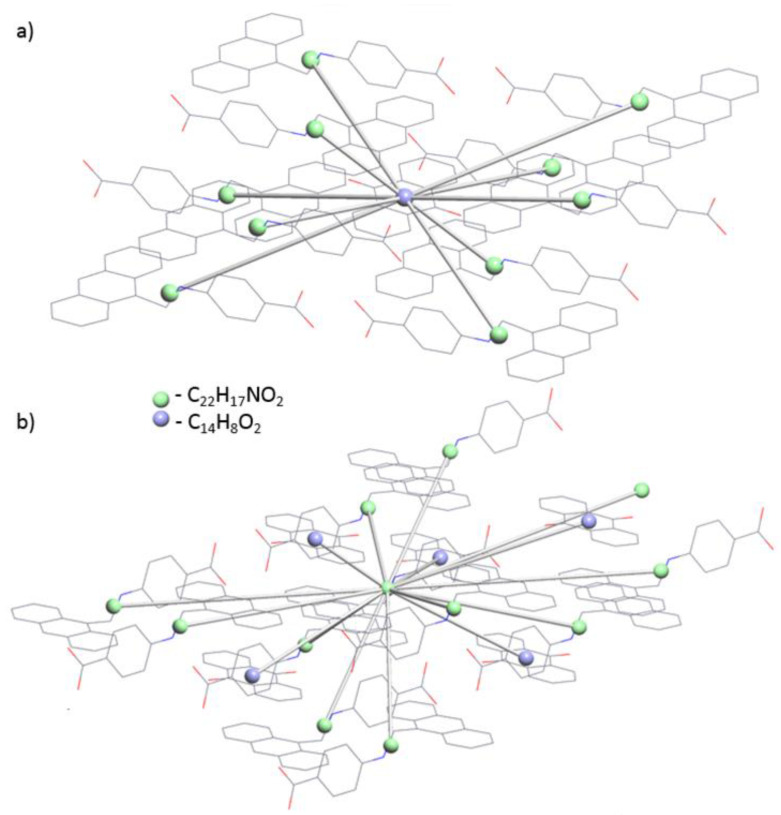
(**a**) C_14_H_8_O_2_ (**top**) and (**b**) C_22_H_17_NO_2_ (**bottom**) molecular environments that are determined in the underlying net in the standard representation of the Coulomb or vdW-bonded molecular structure of L3. Hydrogen atoms were omitted for clarity.

The standard representation of the Coulomb or vdW-bonded structure resulted in the 10,16-c nodal net of the new topological type with a point symbol for the net of: {3^24^.4^19^.5^2^}{3^42^.4^61^.5^17^}_2_ (Figure 4). The details of the underlying net were obtained using the ToposPro software version: 5.4.3.0. This enabled us to know the orientation of the molecules including atoms precisely. Hence, multilevel analysis revealed the structure lying beneath the net through weak or electrostatic interactions; thus, we obtained the following order of the subnets that describe the packing of the structure of complex L3 (Supplementary Materials, Table S6).

### 3.3. Optimized Geometrical Parameters

**Figure 5:**
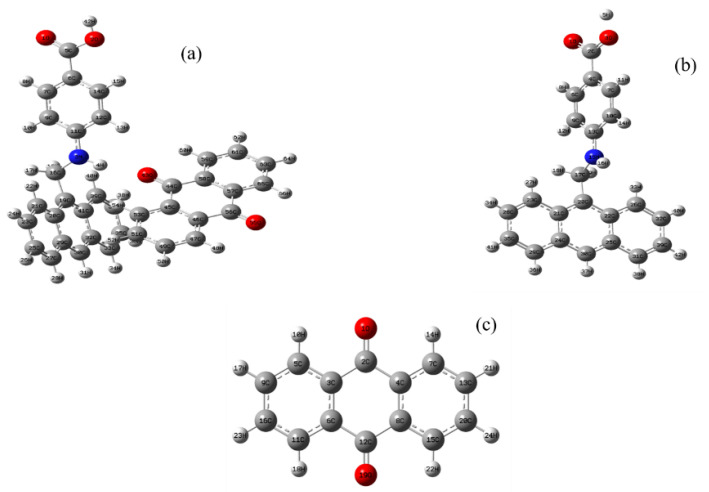
Optimized molecular geometrical structure of (**a**) L3, (**b**) L2 and (**c**) Anthraquinone.

For analyzing the minimum energy configuration of the molecule, an optimization of geometry technique was used. Before exploring a lower-energy geometry, the technique estimates the wave function and energy at an initial geometry. Optimized molecular parameters of L3 in the form of bond length and bond angles are summarized in Supplementary Materials, Table S7 and structures of L3, L2 and anthraquinone are depicted in Figure 5a–c. The optimized molecular parameters of the compounds were obtained via the method B3LYP, with a basis set to 6-311++G(d,p), and theoretical parameters were compared with the experimental single-crystal X-ray diffraction data. L3 possessed C1 point symmetry. The optimized bond parameters were in excellent agreement with XRD results. Additionally, the experimental bond length RMSD value was 0.989 and R^2^ was 0.982. Similarly, the angle RMSD was 0.954 and R^2^ was 0.961. The L3 was a donor–acceptor (D–A) complex with the oxygen of anthraquinone acting as acceptor and the nitrogen of the L2 ligand as donor phenyl ring. There were many phenyl rings in the complex, where C–C lengths were smaller than normal C–C lengths, as C6–C7 was 1.40 Å and C5–C6 was 1.47 Å due to presence of partial double-bond character in the phenyl ring. Carbonyl bond lengths varied depending on their bonding, as O1–C5 was 1.22 Å and O2–C5 was 1.36 Å, due to presence of a double bond in the former and a single bond in the later. The N–H bond length was 1.01 Å and the O–H bond length was 0.97 Å. H4–O43 was 2.09 Å, which was a partial bond due to charge transfer in between the donor and acceptor. The C5–O2–H42 bond angle was calculated to be 105.89°. The N3–H4–O43 bond angle was calculated to be 169.99° and 155.39° experimentally; this angle is between the donor and acceptor molecules, indicating sufficient charge transfer. The O1–C5–O2 bond angle was calculated to be 120.78° and 122.09° experimentally, which is the COOH group angle. Because of the similarities in their environments, the bond lengths of the C–C and C–H bonds remained constant based on the above parameters.

### 3.4. PXRD Studies and TGA Analysis

The PXRD patterns of the as-synthesized and simulated complexes were in agreement, which confirmed the purity of the bulk sample L3 (Supplementary Materials, Figure S2a). The sharp and well-defined peaks illustrated that the crystalline nature of the complex was a result of the formation of new donor–acceptor complexes via charge transfer, in accordance with other spectral studies.

The thermogravimetric analysis of the complex L3 shows two dips throughout the scan. The first endothermic peak at around 230.8 °C may be due to the loss of H-bonding between the two moieties resulting in the loss of the L2 moiety. The second dip in the curve found near about 500 °C is due to the loss of the anthraquinone component, thus making the curve flattened (Supplementary Materials, Figure S2b).

### 3.5. Photocatalytic Activity: Experimental Approach

**Figure 6:**
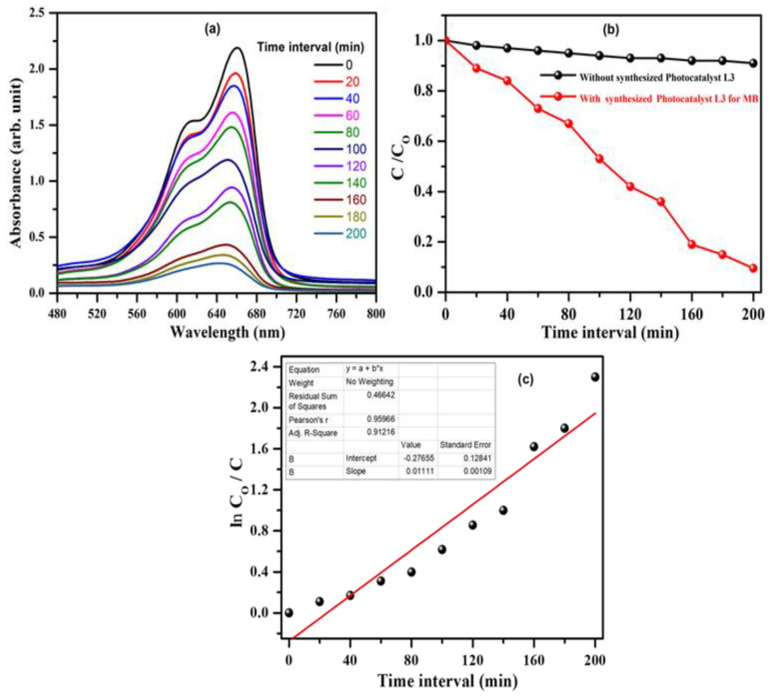
(**a**) Time-dependent UV–Vis spectra of MB dye in the presence of the photocatalyst L3 under the UV light irradiation. (**b**) Time-dependent photocatalytic activity C/C_0_ plot of MB dye in the presence of L3 as photocatalyst. (**c**) Kinetics of dye degradation for the first-order linear plot of lnC/C_0_.

The photocatalytic activity of the synthesized complex (L3) was successfully explored regarding the exposure of UV light towards efficient MB dye degradation. MB is widely used in different industries, displaying the strongest photon absorption at 663 nm. By observing moderate changes in its characteristic peak, i.e., λ_max_ at 663 nm of MB dye with regard to time, the reaction rate of degradation of dye was successfully examined. Figure 6a displays typical time-dependent UV–Vis spectra of decomposed MB dye. Interestingly, there were moderate decreases in the intensity of λ_max_ at 663 nm with respect to time, which confirmed the MB degradation reaction. Figure 6b reveals the extent of MB dye discoloration with successive time intervals in the absence and presence of L3. The efficiency of MB dye degradation can be measured by using the following relation:

Efficiency of degradation (%) = (C_0_ − C/C_0_) × 100 = (A_0_ − A/A_0_) × 100

where C_0_ and C were concentrations of MB dyes solution at time t = 0 and time t = t, and A_0_ corresponds to initial absorbance, whereas A belongs to time-dependent absorbance, respectively. From the degradation exploration graph, it was observed that there was almost negligible amounts of degradation of MB dye in the absence of L3 under prolonged illumination of light, whereas appreciable degradation of MB dye was measured at almost 90% in a time span of just 200 min under similar conditions when the dye degradation reaction was mediated with L3, which confirmed that L3 worked as an efficient photocatalyst for MB dye. The enhanced photodegradation of MB dye mediated with L3 can be described by efficient charge transfer between the donor and the acceptor moiety of L3, which lowers the risk of recombination of photogenerated electron–hole pairs. Figure 6c represents the kinetics of MB dye degradation which apparently follows pseudo-first-order kinetics in accordance with the Langmuir–Hinshelwood mechanism:

Rate = r = −dC/dt = k_L-H_ K_ad_ C/1 + K_ad_ C

where r is the date of MB degradation, C is concentration of MB dye at time t, K_ad_ is the adsorption coefficient of MB dye with the photocatalyst (L3) and k_L-H_ is the rate constant of reaction. The equation for very dilute solutions can be written as:

ln(C_0_/C) = k_L-H_ K_ad_ t ≈ k_app_ t

A plot between ln(C_0_/C) and time offers a straight line with slope k_app_ constant of 0.0111 min^−1^ and with a regression coefficient R^2^ of 0.9596, and the results were inconsistent with previously reported work for other photocatalysts [26,27,28].

As the dye-degradation reaction followed the pseudo-first-order kinetic, the half-life time (t½) of the reaction, i.e., the time at which C = 0.5 and C_0_, can be calculated by using the following equation: t½ = ln2/k, was equal to 63 min, which implies that L3 was an effective photocatalyst [29].

**Scheme 2:**
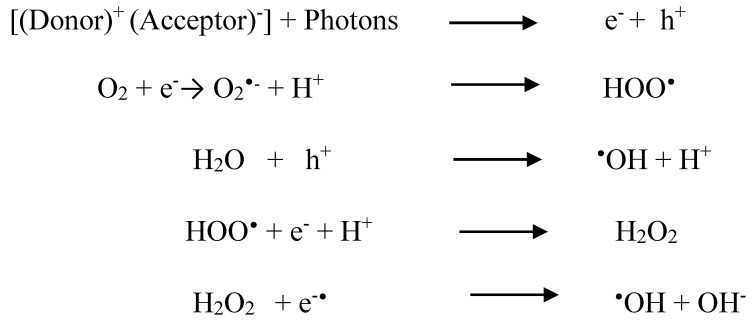
Dye-degradation reaction steps.

**Figure 7:**
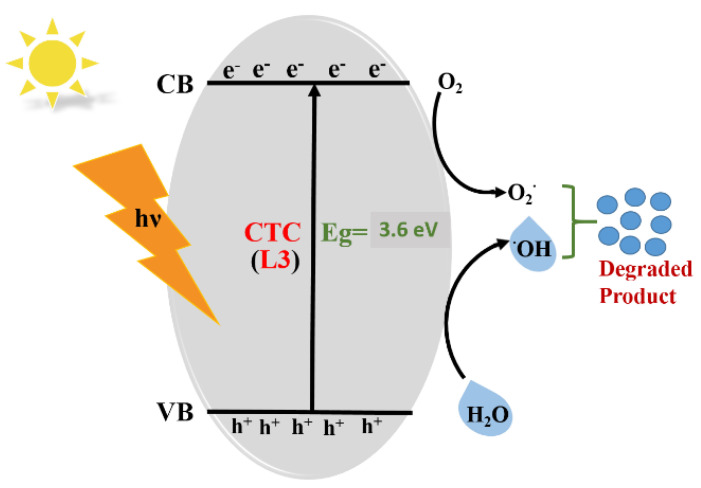
Plausible mechanism of MB dye degradation in the presence of photocatalyst L3.

The mechanism of photocatalytic degradation of MB dyes by donor–acceptor complexes could be explored in view of semiconductor literatures, which explore the advanced oxidation process initiated by hydroxyl radicals (OH^•^) [30]. Figure 7 reveals the plausible mechanism; as the aqueous MB dye suspensions were illuminated with photons bearing the appropriate wavelength, there was promotion of electrons from the highest occupied molecular orbital (HOMO) of the donor moiety to the lowest unoccupied molecular orbital (LUMO) of the acceptor moiety. Subsequently, photogenerated e- moved towards the surface of the L3 complex, and became scavenged by ubiquitous O_2_ to generate superoxide anion O_2_^−^ which became protonated to produce HOO^•^ radicals. However, the unstable radical further reacted with e- and, after a series of reactions, produced a highly active species, H_2_O_2_. Simultaneously, holes (h^+^) moved towards the backside of the photocatalyst surface (L3) and reacted with H_2_O/OH^−^ afterwards with dye molecules to produces active oxidizing species such as the OH^•^ active dye+ molecule (Scheme 2). The following steps could be possible in degradation of dye [31].

Finally, these highly active oxidizing species, i.e., ^•^OH, HOO^•^, OH^−^ and H_2_O_2_, etc. led a series of reactions which decompose MB^+^ dye into less harmful byproducts. Facile charge separation between the [(Donor)^+^(Acceptor)^−^] moiety of complex L3 and the weak interaction with dye was responsible for efficient degradation. There were five different intermolecular attractions, viz., H…N, H…O, H…H, H…C and C…C (π–π), which were responsible for stabilization and fetch of charge between complex L3, as confirmed by 2D fingerprint plots of Hirshfeld surfaces analysis [32]. The anthraquinone had a stable aromatic structure because, with the L2 donor–anthraquinone acceptor complex L3 excited by UV light illumination, there was efficient charge transfer and stabilization. The electron-deficient nature of MB^+^ dye facilitated the interaction between nucleophilic aromatic rings of anthraquinone of the L3 complex and propagated the dye-degradation reaction, which diminished the changes of recombination of charges and enhanced the efficiency of the reaction [33,34].

### 3.6. Mechanism Associated with MB Dye Degradation: Theoretical Approach

**Figure 8:**
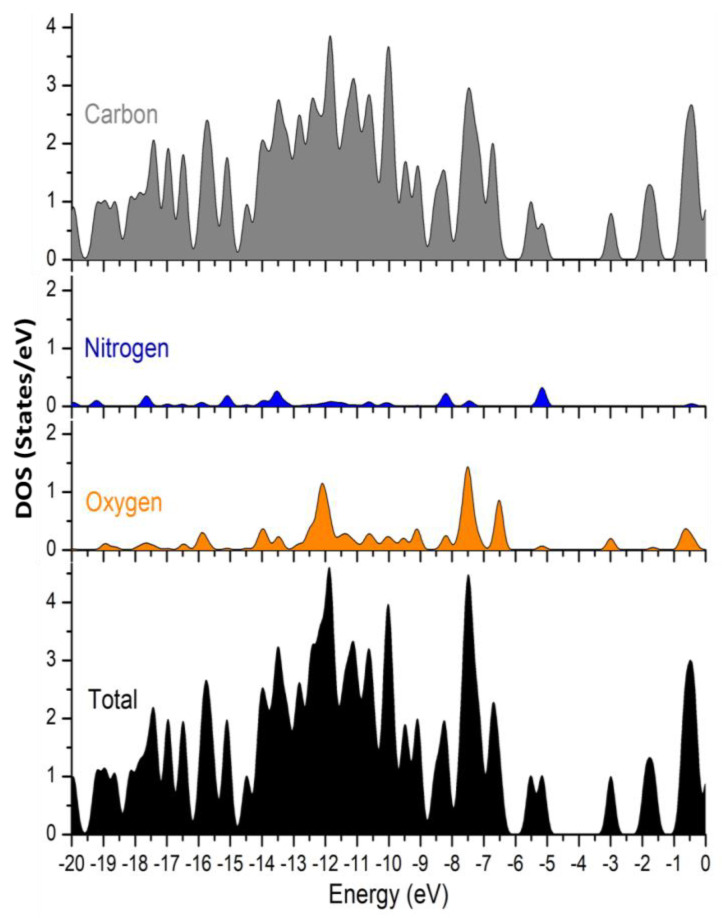
Theoretical band gaps for L3 showed in terms of DOS and p-DOS plots.

Using band-structure calculations, we can easily explain the photocatalytic activity involved with L3. We plotted DOS and p-DOS graphs of L3 with the help of DFT calculations (Figure 8). The results obtained indicated that aromatic O, N and C centers have an important role in contributing to their VB (valence band). The maximum contribution is due to the carbon and oxygen atoms, which suggests that electrons can be transferred from the moiety of L3 to another moiety of L3 involving C and O atoms. As an outcome, the DOS and p-DOS data show that the electronic transition in L3 is primarily of the ligand–ligand type. When light falls on L3, electrons from VB are transferred to CB, leaving equivalent amounts of holes in VB. The moisture in the process can interact with these holes to create ^•^OH radicals, which can break the MB dye. The electrons in the CB, on the other hand, combine with the molecular oxygen superoxide radical that is highly reactive and degrades the organic dye substantially. Less band-gap energy is attributed to photocatalytic efficiency with easier electron promotion from VB to CB, resulting in a higher number of holes being formed in the VB, which are accountable for greater numbers of ^•^OH radicals being produced in their VBs. The higher the number of electrons in the CB, the more superoxide radicals are generated. As previously described in the literature, the ^•^O_2_^−^ and ^•^OH radicals are responsible for organic pollutant photodegradation in substances such as MB dye [35].

### 3.7. Vibrational Spectral Study

Important vibrational modes of the L3 complex were measured via FT-IR (experimental) and compared to the theoretically calculated wavenumbers. The experimental spectra were obtained in solid form, whereas theoretically calculated vibrational frequencies were estimated in isolated molecules in the gaseous phase. DFT calculated wavenumbers illustrated discrepancies revised by utilizing the scaling factors 0.961 and 0.96, below and above 3000 cm^−1^, respectively [14]. The absence of imaginary frequencies during computations in all compounds indicated that the optimized molecular shape was placed at the minimum potential location. Experimental and theoretical FTIR spectra of L3 are shown in Supplementary Materials, Figure S3a,b, and Table S8 summarizes the estimated scaled frequency and IR intensity. Some of the important vibrations are described below.

#### 3.7.1. Ring Vibrations

The stretching vibrations, such as C–H, C–C, and C=C, could be seen in the phenyl ring spectra, as well as different bending vibrations, such as C–C–C, H–C–H and C–C–H. The aromatic ring’s C–H stretching ranged from 3000 to 3150 cm^−1^. In the phenyl ring, C–H stretching seemed like a pure mode. In the FT-IR spectra, the equivalent experimental mode was observed at 3110 and 2852 cm^−1^. The ring C–C stretching appeared in a range of 1625 to 1430 cm^−1^. At 1580 cm^−1^, a very sharp band was detected, which was compatible with C–C stretching. The aromatic ring’s C–C bending in-plane occurred as mixed modes with the C–H in-plane bending vibration.

#### 3.7.2. C=O, N–H and O–H Vibrations

A carbonyl group is a polar multiple-bonded group established by p–p bonding between carbon and oxygen. The difference in electro-negativities causes electrons to move towards oxygen, resulting in the carbonyl group. The carbonyl group’s nature is determined by the lone pair of oxygen [36]. In L3, stretching of C=O arose theoretically at 1650, 1618, 1607, 1602, 1560 and 1549 cm^−1^, depending on conjugation, hydrogen bonding, and the size of the ring to which it was attached. Carbonyl groups’ bending modes are likely to be weak and observed as mixed modes. For C=O stretching, FT-IR vibrations were found at 1750 and 1601 cm^−1^. An OH vibrational mode was found at 3490 cm^−1^ and 3351 cm^−1^ experimentally.

N–H stretching peaks arise at 3300–3500 cm^−1^ for single bonds. Because the L3 complex has only one NH group, the peak emerged at 3433 cm^−1^. Bending NH vibrational modes were found as mixed-mode vibrations at roughly 1600 cm^−1^ due to nitrogen–carbon bond stretching and bending of the N–H bond at 1427 cm^−1^ at lower frequencies, i.e., 1641–1085 cm^−1^.

### 3.8. Molecular Electrostatic Potential (MEP)

**Figure 9:**
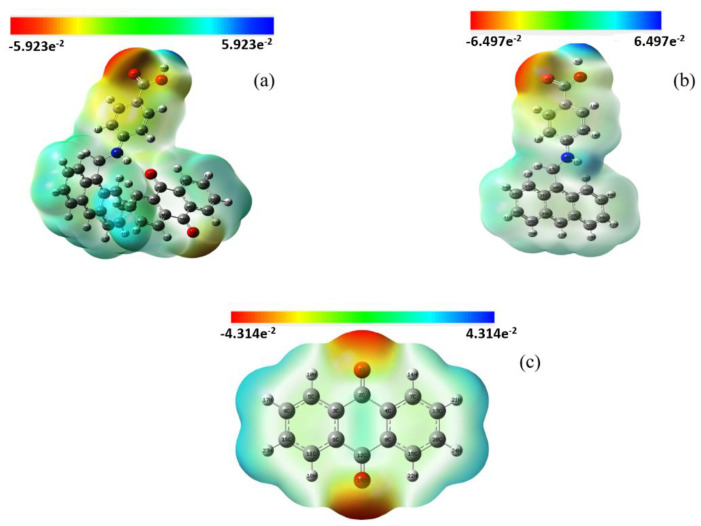
MEP map of the (**a**) L3, (**b**) L2 and (**c**) Anthraquinone.

Figure 9 depicts the strength of electrostatic potentials of the reactant and produced moieties using a molecular electrostatic potential map (MEP) [37]. The electronegative zone is represented by red color regions, whereas the electropositive region is represented by blue color regions. The existence of L2 and anthraquinone as a donor–acceptor moiety can be theoretically explained by the MEP, as an electron-rich region lies at the C=O bond of anthraquinone, and it is electron deficient at the NH of L2, which was observed to be faded when anthraquinone interacts with L2. In L3, L2 and anthraquinone, the highest negative range was −5.923 × 10^−2^, −6.497 × 10^−2^ and −4.31 × 10^−2^, showed in the figure by a red shade and called an electrophilic region. A nucleophilic area was defined as a positive range of +5.923 × 10^−2^, +6.497 × 10^−2^ and +4.31 × 10^−2^, which was represented by a blue tint. The electrostatic potential is modest, as evidenced by the little yellow tint between the phenyl rings. The light blue area (MEP of L3) in between L2 and anthraquinone shows the transfer of electrons from donor to acceptor.

### 3.9. Non-Linear Optical (NLO) Analysis

To determine NLO properties in terms of the calculated polarizability (*α*), hyperpolarizability (*β*) and dipole moment (*µ*) of L3, an isolated molecule in the gas phase of L3 was used [38]. DFT with the B3LYP and 6-311++G(d,p) basis set was used. The polarizability and hyperpolarizibility coefficients (*α_xx_*, *α_xy_*, *α_yy_*, *α_xz_*, *α_yz_*, *α_zz_* and *β_xxx_*, *β_xxy_*, *β_xyy_*, *β_yyy_*, *β_xxz_*, *β_xyz_*, *β_yyz_*, *β_xzz_*, *β_yzz_*, *β_zzz_*) from the Gaussian frequency output file were used. To begin, the units were converted to e.s.u (1 a.u. = 0.1482 × 10^−24^ e.s.u., 1 a.u. = 8.6393 × 10^−33^ e.s.u.) [18]. The dipole moment, polarizability and first-order hyperpolarizability of NLO active materials were all high. Total polarizability (*α*) and the mean value of initial hyperpolarizability (*β*) were calculated using the equations below.

For analyzing polarizability (*α*) and hyperpolarizability (*β*) the following equations were used:

$$\alpha_{tot}=\frac{1}{3}\left(\alpha_{xx}+\alpha_{yy}+\alpha_{zz}\right)\;\beta =[{\left(\beta_{xxx}+\beta_{xyy}+\beta_{xzz}\right)}^{2}+{\left(\beta_{yyy}+\beta_{yzz}+\beta_{yxx}\right)}^{2}+{\left(\beta_{zzz}+\beta_{zxx}+\beta_{zyy}\right)}^{2}]{½}^{\;}$$

Dipole moment $\mu_{i}$, *i* = *x*, *y*, *z*, total dipole moment *μ_tot_* = ($\mu_{x}^{2}$ + $\mu_{y}^{2}$ + $\mu_{z}^{2}$)^½^.

Because of the presence of very strong interactions between the molecules, a high dipole moment value was observed at approximately 6.5518 D along *μ_x_*, as shown in Supplementary Materials, Table S9. Furthermore, the polarizability value estimated using the B3LYP/6-311++G(d,p) technique was −3.4 × 10^−23^ e.s.u, which was lower than the urea value of 0.38312 × 10^−23^ e.s.u. L3 had a first-order hyperpolarizability value of 2.39 × 10^−30^ e.s.u, which was lower than the urea value of 0.3728 × 10^−30^ e.s.u. This suggests that the L3 complex has significant NLO features. As a result, it can be used as an NLO active complex in future studies.

### 3.10. UV–Vis and Electronic Properties

The electronic spectra were reproduced using the TD-DFT/B3LYP approach and 6-311++G(d,p) basis set, which included PCM (polarizable continuum solvation model) with ethanol [39]. Values for λ_max_, excitation energy and band gap are depicted in Table 2. With ethanol as the solvent, the experimental λ_max_ was observed at 252 nm. In ethanol, the calculated theoretical absorption maximum was found at 262 nm. HOMO to LUMO energy band gap can be used to examine the compound’s stability, chemical activity and other characteristics [40].

The UV–Vis absorption spectra of both L2 and L3 were scanned in the range from 200 to 700 nm on a UV–Visible absorption spectrophotometer. The solutions of both were prepared in a 10^−4^ M concentration using polar organic solvent ethanol. The λ_max_ of L2 and L3 were found to be 243.5 nm and 252 nm, respectively (Supplementary Materials, Figure S4a). The shifting of the peak from lower to higher wavelength in the case of L3 as compared to L2 can be attributed to n–π* transitions occurring between L2 (as donor) and anthraquinone (as acceptor). The spectrum of L3 was also produced theoretically by using TD/DFT calculations and was found to be in agreement with the experimental one (Supplementary Materials, Figure S4b).

The following equation was applied to calculate band-gap energy of synthesized materials:

$$\left(ahv\right)=A{\left(hv-E_{g}\right)}^{n/2}$$

where *E_g_*, *ν* and *α* are the band-gap energy, light frequency and absorption coefficient, respectively. *A* is a constant and the value of *n* can be evaluated depending on the type of electronic change. The *E_g_* values for the samples were calculated by extending the curvature to the photon energy in a linear fashion. The graph of (*αhv*)2 vs. *hν* can be found in Supplementary Materials, Figure S5. Complex L3′s band-gap energy was discovered to be 3.5 eV. The computed band-gap energy data suggest that the produced complex has fairly high photocatalytic activity when exposed to UV light.

**Figure 10:**
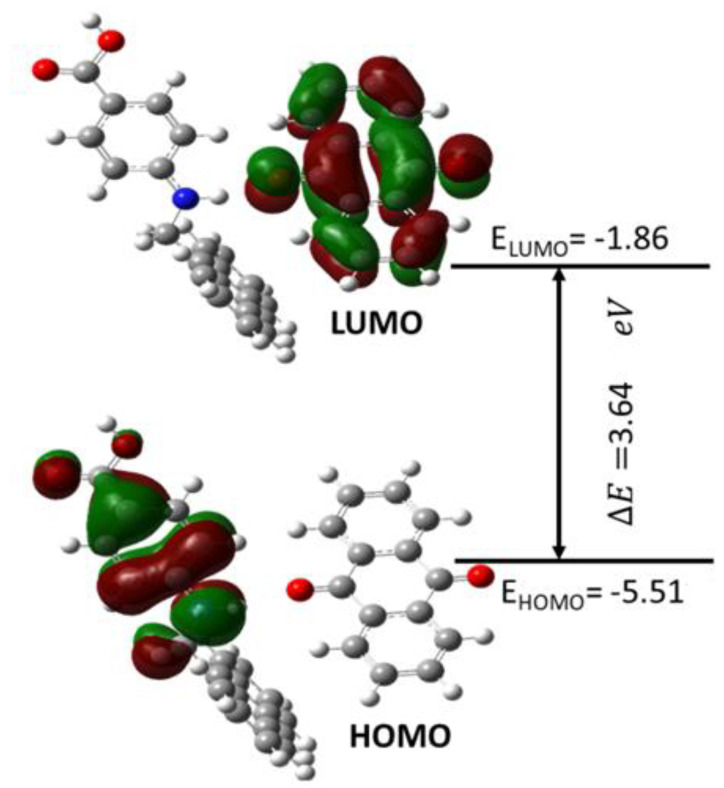
Pictorial representation of frontier molecular orbital showing HOMO–LUMO energy gap.

Figure 10 shows the chemical stability of a molecule which is approximated by the HOMO→LUMO energy gap. The LUMO energy value was −1.86 eV, while the HOMO energy value was −5.51 eV. HOMO–LUMO energy gap values were helpful in determining a number of other important variables, including ionization potential (IE), electron affinity (EA), electronegativity (E), chemical hardness, chemical softness, chemical potential and electrophilicity index (Supplementary Materials, Table S10). This band gap demonstrated that L3 was a stable molecule with bioactive properties and that charge transfer could occur within this molecule. Chemical hardness, in addition to the band gap, provided chemical stability parameters. The complex L3 is chemically stable due to its high chemical hardness value, 1.825. Similarly, the electronegativity value which estimates electron attraction in a covalent bond was estimated to be 3.64. The highest electron flow between HOMO and LUMO resulted in an L3 electrophilicity value of 3.71. (Supplementary Materials, Table S10).

### 3.11. Hirshfeld Surface Analysis

**Figure 11:**
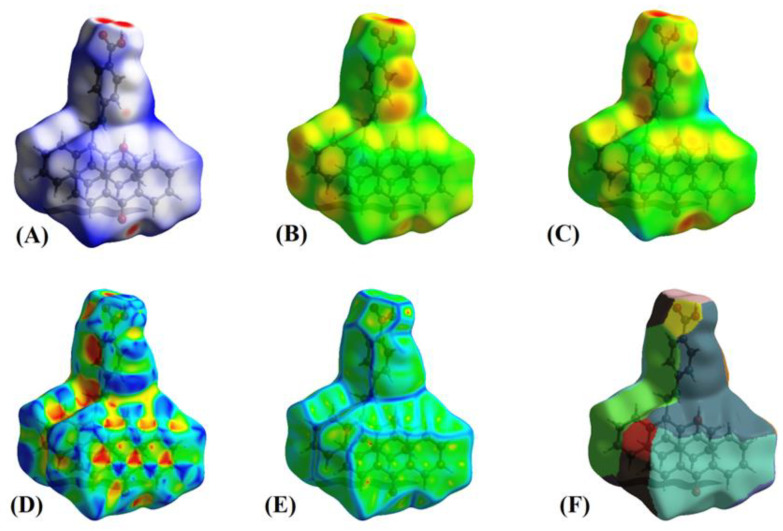
Hirshfeld surface of the complex mapped with (**A**) d_norm_, (**B**) d_i_, (**C**) d_e_, (**D**) shape index (**E**), curvedness and (**F**) Fragment patches.

Interactions between atoms in the crystal of L3 were investigated using Hirshfeld surface analysis. Figure 11 shows the L3 Hirshfeld surface mapped using d_norm_, d_i_, d_e_, shape index, curvedness and fragment patch, respectively. The distance between the atoms existing in the crystal system is described by the d_i_ and d_e_ pair’s sum. Lists of all 3D surfaces with their lowest, maximum, and mean values, as well as their d_norm_, d_i_, d_e_, shape index, curvedness and fragment values are given in Supplementary Materials, Table S11.

In L3, the d_norm_ value ranged from −0.787 to 2.364 Å, the shape index ranged from −0.995 to 0.998 Å, and the curvedness ranged from −3.625 to 0.397 Å, as shown in Supplementary Materials, Table S11. The d_norm_ surface has brick red circular patches, representing close interaction with surrounding molecules; a hydrogen connection between O–H and N–H was discovered despite the fact that the intermolecular H–H, N–H, C–H and S–H interactions are defined by blue and white patches. Because the O atom has a greater electronegativity (3.34) than the N atom (3.04), it is possible that the oxygen atom influenced the electron density on the H atom the most. One of the key characteristics of Hirshfeld analysis is the shape index, which identifies complementarity between molecules in crystal packing [41]. Patches of varied colors on a “shape index” surface exhibiting intermolecular complementarity zones are shown in Figure 11D. The pie-stacked molecules in the concave section are represented by red highlighted patches. The ring structure of the molecule inside the crystal surface is depicted by the convex area in blue.

Curvature is used to determine the surface area of a molecule’s shape [42]. The electrostatic contact between the molecules is represented by the curvature. Figure 11E depicts the curvedness of the L3 complex, which ranges between −3.625 and 0.397. Low curvedness on the surface refers to flat disc-like regions, but high curvedness corresponds to sharp edge-like curvature that is likely to stretch the surface into patches, indicating contact between neighboring molecules. The blue edge that divides flat parts illustrates π–π stacking interactions. Two-Dimensional fingerprint pictures reflect the type of intermolecular interactions between atoms, as well as the variations in these patterns, and they are a significant intermolecular link in overall crystal structure [42]. Fingerprint plot specifically reveals that all components of the molecule are in close proximity. Two-Dimensional fingerprint plots of distinct intermolecular interactions of L3 and corresponding d_norm_ Figures, along with the proportion of intermolecular contacts provided by each atom are available in Supplementary Materials, Figure S6.

These 2D plots revealed small spikes, such as a pseudo-symmetrical region with a high concentration of blue color, that encircled the entire area contributed [41]. Almost all of the exchanges listed above provide information about the 3D network of the L3 complex [42]. The enrichment ratio was determined to confirm the accuracy of the data on the contribution of bonds produced in the molecule. Because of the enormous amount of hydrogens on the molecular surface, H…H contacts were the most stable (around 47.4%) in L3. In L3, according to calculations, H–H bonds created 47.4% of the molecular surface, accompanied by C–H with 29.4% and O–H with 17.6%, as shown in Supplementary Materials, Table S12.

### 3.12. Energy Framework Calculations

**Figure 12:**
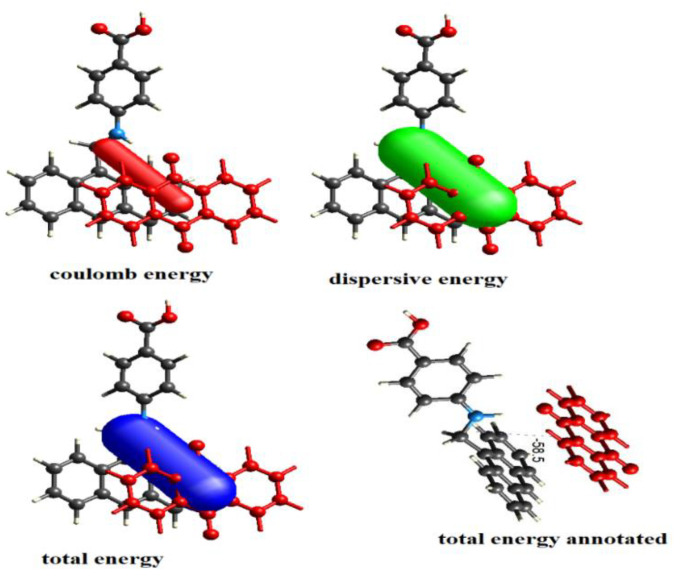
Graphical representation of electrostatic interactions, coulomb interaction energy, dispersion energy and total energy of the CT complex.

The TONTO program [43], which is built into Crystal Explorer 17 software, was employed to determine the energy framework and interaction energies of the CT complex. B3LYP/6-311G(d,p) energy model available in Crystal Explorer with scale factors k_ele = 1.057, k_pol = 0.740, k_disp = 0.871 and k_rep = 0.618, respectively [44] was used to estimate the intermolecular interaction energies for L3. The different interaction energies coulombic interaction energy (red), dispersion energy (green) and total interaction energy (blue) of L3 are depicted in Figure 12. The magnitude of the interaction energy is proportional to the radii of the corresponding cylinder. Table 3 shows the result of different interaction energies of L3. The calculated electrostatic, polarization, dispersion and repulsion energies are −25.9 kJ/mol, −5.3 kJ/mol, −71.2 kJ/mol and 56.5 kJ/mol, respectively, and −58.5 kJ/mol is the calculated total energy of the molecule. The result of this study illustrated that dispersion energy was predominant over the other interaction energies, playing a vital role in total forces in crystal packing.

### 3.13. Molecular Docking

**Figure 13:**
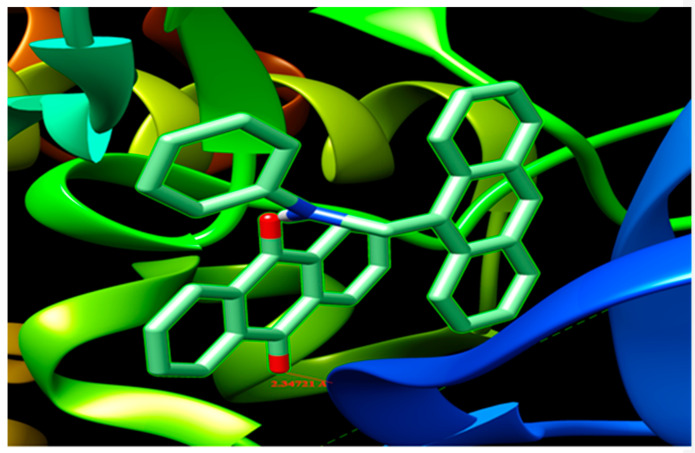
Docked pose of L3 embedded in the active sites of 5E1S protein.

Molecular docking [45] is the most common technique for studying the relationship of structure and activity of the ligand with protein as drug in biological applications. It discloses the strength of the interaction between a small molecule ligand and its macromolecular targets (receptor), as well as its binding energy and binding location [46]. It plays a pivotal role in pharmaceutical research and development since it examines key molecular actions [47]. Autodock Vina [48] is a molecular docking application that is free and open source. From the Swiss ADME-Target prediction site, the suitable target protein ID was chosen and retrieved from the protein data bank (PDB). The oxidoreductase protein 5E1S was docked with the donor–acceptor complex L3, as shown in Figure 13, with a bond distance of 2.34. Figure 13 depicts electrostatic interactions in terms of H-bonding between L3 and the target protein 5E1S. The binding energy of the ligand and the receptor protein is −10.8 kcal/mol (Supplementary Materials, Table S13), with the H-bond between the residue and the ligand determining the ligand’s and receptor protein’s stability. The low binding energy value acquired suggested that the examined chemical is physiologically active. In the protein, there were three residues.

## 4. Conclusions

Herein, we report a novel donor–acceptor complex, L3, synthesized using anthraquinone and our own synthesized ligand (L2) via reflux method. The synthesized complex (L3) was characterized through different techniques including SCXRD, FTIR, electronic spectroscopy, PXRD and TGA. The SCXRD results revealed that the formation of new L3 complex occurred via H-bonding between the donor (L2) and acceptor moiety (anthraquinone). We also performed theoretical studies including Hirshfeld surface analysis and DFT/TD-DFT to decode the surface and short interactions. The DFT/TD-DFT calculations showed that optimized geometrical parameters, FTIR and UV spectra of L3 were in agreement with the experimental results. NLO was evaluated, and total dipole moment was observed to be 6.572 D. NLO studies suggested that the L3 complex can be considered a high quality NLO material. The frontier molecular orbitals HOMO and LUMO were located in their spatial arrangements and the energy gap (∆E) was found to be 3.6463 eV. With the help of these calculations, we successfully employed L3 as a photocatalyst in the degradation of Methylene blue (MB) dye. Almost 90% of dye was degraded in just 3.2 h. Computed results of density of state and partial density of states assisted the plausible mechanistic pathways by which L3 accomplished photodegradation. The results were promising for the use of L3 as a photocatalyst in the degradation of other dyes as well in future applications. Additionally, molecular docking revealed that the lowest binding energy was −10.8 Kcal/mol, which indicates that the L3 complex may be further studied for its medicinal applications.

## Figures and Tables

**Table 1:** Crystallographic refinement data and refinement parameters for L3.

CCDC Number	2102211
Empirical formula	C_29_H_21_NO_3_
Formula weight	431.49
Temperature/K	100(2)
Crystal system	Triclinic
Space group	P-1
a/Å	8.1901(7)
b/Å	12.4958(10)
c/Å	12.5930(11)
α/°	64.784(2)
β/°	82.636(2)
γ/°	77.491(2)
Volume/Å^3^	1137.36(17)
Z	2
ρcalcg/cm^3^	1.2599
μ/mm^−1^	0.082
F(000)	452.2
Crystal size/mm^3^	0.48 × 0.27 × 0.15
Radiation	Mo Kα (λ = 0.71073)
2Θ range for data collection/°	6.08 to 50.1
Index ranges	−10 ≤ h ≤ 10, −16 ≤ k ≤ 16, −16 ≤ l ≤ 16
Reflections collected	16,550
Independent reflections	4008 [R_int_ = 0.0399, R_sigma_ = 0.0471]
Data/restraints/parameters	4008/1/302
Goodness-of-fit on F2	1.063
Final R indexes [I> = 2σ (I)]	R_1_ = 0.0512, *wR*_2_ = 0.1153
Final R indexes [all data]	R_1_ = 0.0629, *wR*_2_ = 0.1215
Largest diff. peak/hole/e Å^−3^	0.32/−0.28

**Table 2:** Comparison of electronic properties of L3 attained experimentally and calculated by using the TD-DFT/B3LYP method.

Solvent	TD/DFT
λ_max_ (nm)	Band gap (eV)
Experimental	
Ethanol (L3)	
252	4.92
Ethanol (L2)	
243.5	5.09
Calculated	
Ethanol	
262	4.73
241	5.14

**Table 3:** Different interaction energies of compound in KJ/mol.

Energy Model	k_ele	k_pol	k_disp	k_rep
B3LYP/6-311G(d,p) electron densities	1.057	0.740	0.871	0.618
Electron Density	E_ele	E_pol	E_dis	E_rep
B3LYP/6-31G(d,p)	−25.9	−5.3	−71.2	56.5

## Data Availability

Not applicable.

## Supplementary Materials

The following are available online: The details of X-ray crystal refinement, selected bond lengths [Å], bond angles [°], fractional atomic coordinates, anisotropic displacement parameters, Hydrogen atom coordinates and Topological analysis details are given in Tables S1–S6. Optimized geometrical parameters, calculated vibrational frequencies, values of calculated dipole moment μ(D), polarizability (α_0_) and first-order hyperpolarizability are given in Tables S7–S9. All energy calculated values including ionization potential, electron affinity, electronegativity, etc., are listed in Table S10. The Hirshfeld surface analysis and molecular docking details are given in Tables S11–S13, respectively. Figure S1 shows molecular fragments of L3; Figure S2a shows the simulated and as-synthesized PXRD; Figure S2b shows the TGA analysis curve. Figure S3a,b shows experimental and simulated FTIR spectra for the L3 complex, respectively. Figure S4a,b shows UV–V is spectra for the L3 complex while Figure S5 shows the Tauc plot for the same. The 2D surfaces plotted for Hirshfeld surface analysis of the L3 complex are shown in Figure S6. References [49,50,51,52,53,54,55,56,57,58,59,60,61,62,63,64,65,66,67,68,69] are cited in the Supplementary Materials.

## Sample Availability

Samples of the compounds are available from the authors.
