# Supplementary material for: Crystal Structure, Topology, DFT and Hirshfeld Surface Analysis of a Novel Charge Transfer Complex (L3) of Anthraquinone and 4-{[(anthracen-9-yl)meth-yl] amino}-benzoic Acid (L2) Exhibiting Photocatalytic Properties: An Experimental and Theoretical Approach
Source: Molecules. 2022 Mar 6;27(5):1724. doi: 10.3390/molecules27051724 (PMC8912118; doi:10.3390/molecules27051724)
Supplement: Supplementary file 1 [file molecules-27-01724-s001.zip › molecules-1610021-supplementary.pdf]

# Supplementary Information

## 2. Experimental

*2.1. X-ray crystal refinement*

The diffraction data was collected with a Bruker SMART APEX CCD diffractometer using monochromatic Mo-K$\alpha$ radiation ($\lambda$=0.71073 Å) at 100 (2) K. The linear absorption coefficients and the anomalous dispersion corrections were mentioned from the International Tables for X–ray crystallography [1]. Using Olex2, the structure was solved with the olex2.solve [2] structure solution program using Charge Flipping and refined with the olex2.refine [2] refinement package using Gauss-Newton minimisation. All hydrogen atoms were located in difference Fourier maps in the structures and refined isotropically. All non–H atoms were refined anisotropically.

*2. 2. Topological Analysis*

The analysis was performed with the topcryst.com [3]. The RCSR three-letter codes [4] were used to designate the network topologies. Those nets, that are absent in the RCSR, are designated with the TOPOS NDn nomenclature [5], where N is a sequence of coordination numbers of all non-equivalent nodes of the net, D is periodicity of the net (D=M, C, L, T for 0-,1-,2-,3-periodic nets), and n is the ordinal number of the net in the set of all non-isomorphic nets with the given ND sequence. To calculate the underlying nets, we used algorithms [5], the application of which for specific structures is discussed in the article [6]. The TTD collection [7] was used to determine the topological type of the crystal structure.

*2.3. Computational details*

Among the numerous quantum-computing techniques, the Density Functional Theory (DFT) is a useful tool for describing key molecular characteristics. The B3LYP approach and 6-311++G(d,p) basis set [8,9] supplied in the Gaussian 03W and Gauss View [10] were used for the most of the computational calculations, and some of the findings were also acquired via ORCA (4.0.1) [11]. Geometrical bond parameters, vibrational wave numbers, HOMO-LUMO energy gap and molecular electrostatic potential (MEP) are molecular properties that were all achieved by the optimized molecular structure. The Swiss ADME tool [12] provides a molecule with an active substance-like property, which is required for synthesis of active compounds. Visualization was done with Multiwfn software [13], graph plotting was carried out by Origin8.0 programme [14]. IP and EA were estimated using Koopman's theorem, i.e. EA= - ELUMO and IP= -EHOMO [15] and chemical reactivity descriptors were derived using IP and EA by adopting the formulas given below.

Electro-negativity ($\chi$) = $\frac{IA+EA}{2}$, Chemical potential ($\mu$) = $\frac{-(IA+EA)}{2}$

Chemical hardness ($\eta$) = $\frac{-IA-EA}{2}$

Chemical softness ($\sigma$) = = $\frac{1}{2\eta}$, Electrophilicity index ($\omega$) = $\frac{\mu*\mu}{2\eta}$

Hirshfeld surface analysis was used to determine the intramolecular and intermolecular interconnections inside the crystal surface, as well as the H-bonded interactions. It is a useful technique for analysing unit cell packing in crystal. The study is based on a 3-D graphical representation in the geographical region where the molecule interacts with nearby molecules, and 2-D fingerprint images that describes the kind of atomic interaction. This analysis was used to study crystals qualitatively and quantitatively [16,17]. The Crystal Explorer 17 software [18] was applied to record the Hirshfieldsurface and fingerprint. Eq. [19] may be used to determine the mapping of the normalised contact (dnorm).

$$d_{norm}=\frac{d_i-r_i^{vdw}}{r_i^{vdw}}+\frac{d_e-r_e^{vdw}}{r_e^{vdw}}$$

$r_i^{vdw}$ and $r_e^{vdw}$ represents the radii of atoms (van der waals). The distance from the surface to the closest nucleus di (internal) and de (external) to the atom were used to analyze the interactions in crystals. 3-D Hirshfeld surface images rendered in the Hirshfeld surface as dnorm, with a red colour area corresponding to negative dnorm side and a near intermolecular interaction, and a blue color area corresponds positive dnorm value and longer intermolecular interactions. The zero dnorm value is represented by the white portion. The di and de ends in the 2-D fingerprint diagram indicate a sort of intermolecular interactions that exists inside the crystal's molecules [20].

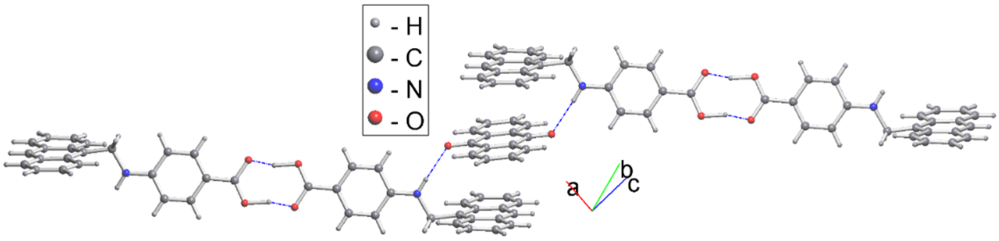


**Figure S1.** The structural fragments of molecular structure of L3

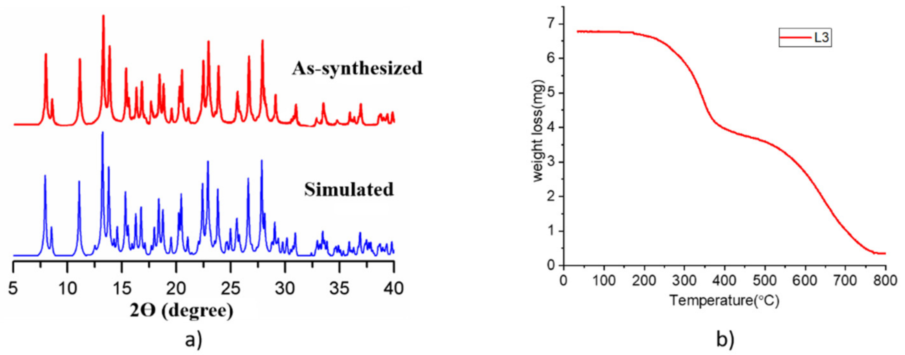


**Figure S2. (a)** Simulated and As-synthesized PXRD of L3, **(b)** TGA curve of L3

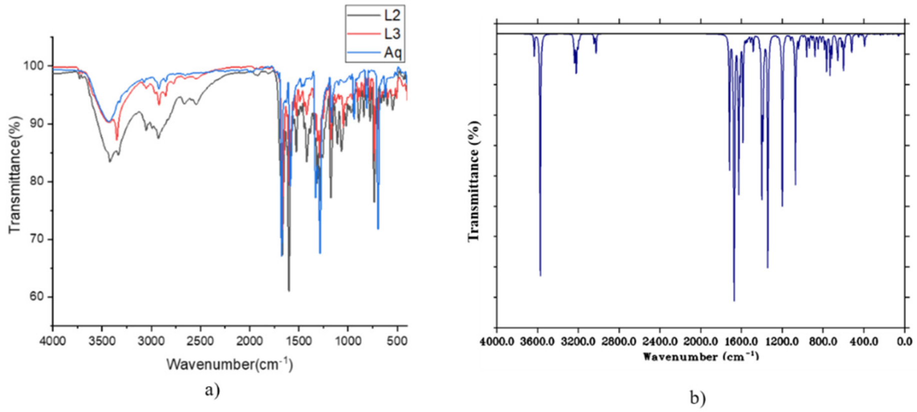


**Figure S3** (a) Experimental FTIR spectra of L2, L3 and Anthraquinone, (b) Simulated FTIR spectrum of L3

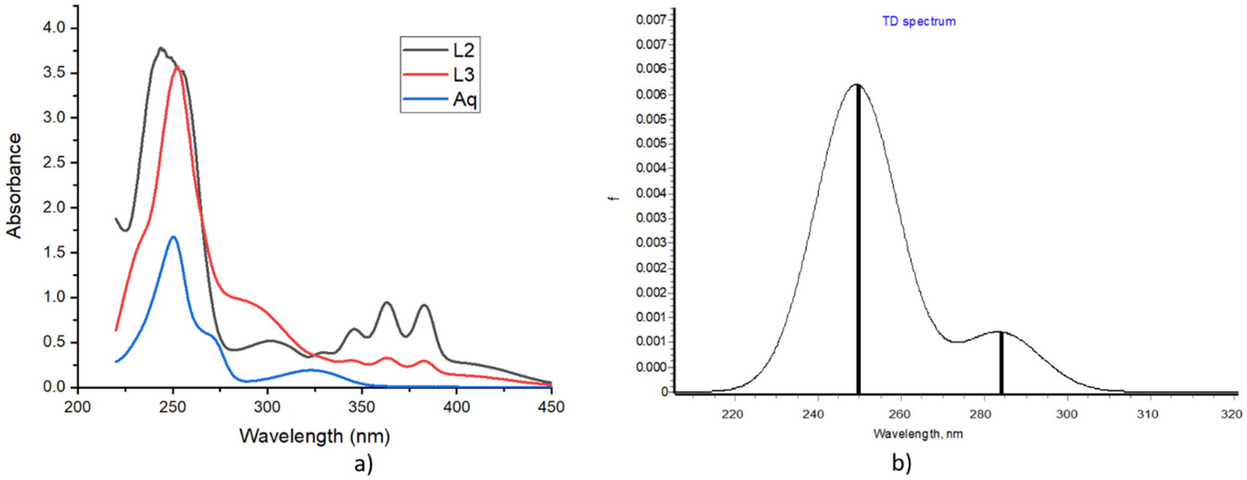


**Figure S4** (a) Experimental UV-Visible spectra of L2 and L3, (b) UV-visible spectrum of L3 obtained through TD-DFT calculations

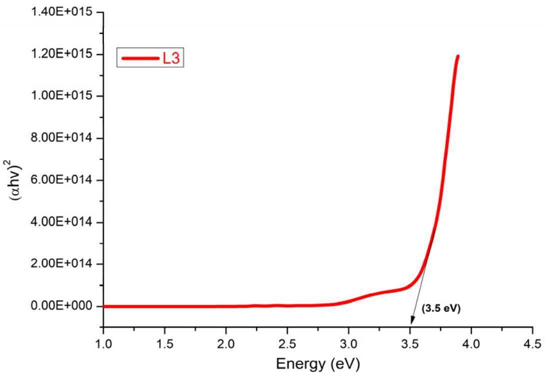


**Figure S5.** Tauc's plot showing band gap

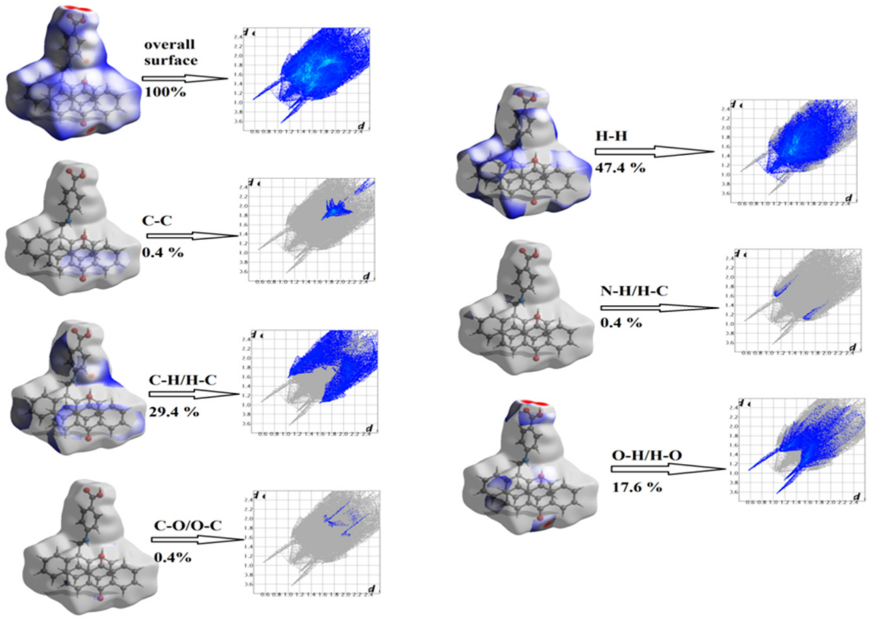


**Figure S6.** 2-D fingerprint plot of different dnorm contributions for L3

**Table S1**. Bond Lengths for L3

| Atom | Atom | Length/Å | Atom | Atom | Length/Å |
|---|---|---|---|---|---|
| O1 | C1 | 1.280(2) | C13 | C14 | 1.353(3) |
| O2 | C1 | 1.277(2) | C14 | C15 | 1.426(3) |
| O3 | C23 | 1.225(2) | C15 | C16 | 1.391(3) |
| N1 | C5 | 1.357(2) | C16 | C17 | 1.390(3) |
| N1 | C8 | 1.453(2) | C17 | C18 | 1.429(3) |
| C1 | C2 | 1.464(2) | C17 | C22 | 1.439(3) |
| C2 | C3 | 1.395(3) | C18 | C19 | 1.351(3) |
| C2 | C7 | 1.404(3) | C19 | C20 | 1.420(3) |
| C3 | C4 | 1.371(3) | C20 | C21 | 1.357(3) |
| C4 | C5 | 1.412(3) | C21 | C22 | 1.431(3) |
| C5 | C6 | 1.413(3) | C23 | C24 | 1.487(3) |
| C6 | C7 | 1.370(3) | C23 | C29[1] | 1.480(3) |
| C8 | C9 | 1.509(2) | C24 | C25 | 1.388(3) |
| C9 | C10 | 1.410(3) | C24 | C29 | 1.404(3) |
| C9 | C22 | 1.407(3) | C25 | C26 | 1.382(3) |
| C10 | C11 | 1.436(3) | C26 | C27 | 1.384(4) |
| C10 | C15 | 1.436(3) | C27 | C28 | 1.372(3) |
| C11 | C12 | 1.356(3) | C28 | C29 | 1.394(3) |
| C12 | C13 | 1.417(3) | | | |

[1]1-X,1-Y,1-Z

**Table S2.** Bond Angles for L3

| Atom | Atom | Atom | Angle/˚ | Atom | Atom | Atom | Angle/˚ |
|---|---|---|---|---|---|---|---|

| | | | | | | | |
|---|---|---|---|---|---|---|---|
| C8 | N1 | C5 | 121.88(15) | C16 | C15 | C10 | 118.91(18) |
| O2 | C1 | O1 | 122.25(17) | C16 | C15 | C14 | 121.98(18) |
| C2 | C1 | O1 | 118.96(16) | C17 | C16 | C15 | 122.29(18) |
| C2 | C1 | O2 | 118.79(16) | C18 | C17 | C16 | 121.95(18) |
| C3 | C2 | C1 | 121.49(16) | C22 | C17 | C16 | 119.04(18) |
| C7 | C2 | C1 | 120.08(16) | C22 | C17 | C18 | 118.99(18) |
| C7 | C2 | C3 | 118.43(17) | C19 | C18 | C17 | 121.44(19) |
| C4 | C3 | C2 | 121.31(17) | C20 | C19 | C18 | 119.90(19) |
| C5 | C4 | C3 | 120.37(17) | C21 | C20 | C19 | 120.8(2) |
| C4 | C5 | N1 | 122.25(16) | C22 | C21 | C20 | 121.61(19) |
| C6 | C5 | N1 | 119.39(16) | C17 | C22 | C9 | 119.55(17) |
| C6 | C5 | C4 | 118.35(17) | C21 | C22 | C9 | 123.24(17) |
| C7 | C6 | C5 | 120.38(17) | C21 | C22 | C17 | 117.20(17) |
| C6 | C7 | C2 | 121.15(17) | C24 | C23 | O3 | 121.0(2) |
| C9 | C8 | N1 | 112.13(15) | C29[1] | C23 | O3 | 120.6(2) |
| C10 | C9 | C8 | 119.43(17) | C29[1] | C23 | C24 | 118.33(18) |
| C22 | C9 | C8 | 120.24(17) | C25 | C24 | C23 | 120.0(2) |
| C22 | C9 | C10 | 120.25(17) | C29 | C24 | C23 | 120.57(19) |
| C11 | C10 | C9 | 122.80(18) | C29 | C24 | C25 | 119.5(2) |
| C15 | C10 | C9 | 119.78(18) | C26 | C25 | C24 | 120.3(2) |
| C15 | C10 | C11 | 117.42(18) | C27 | C26 | C25 | 120.1(2) |
| C12 | C11 | C10 | 121.2(2) | C28 | C27 | C26 | 120.3(2) |
| C13 | C12 | C11 | 120.9(2) | C29 | C28 | C27 | 120.5(2) |
| C14 | C13 | C12 | 120.06(19) | C24 | C29 | C23[1] | 121.09(19) |
| C15 | C14 | C13 | 121.3(2) | C28 | C29 | C23[1] | 119.6(2) |
| C14 | C15 | C10 | 119.09(18) | C28 | C29 | C24 | 119.3(2) |

[1]1-X,1-Y,1-Z

**Table S3.** Fractional Atomic Coordinates (×10[4]) and Equivalent Isotropic Displacement Parameters (Å$^2$×10$^3$) for L3. $U_{eq}$ is defined as 1/3 of of the trace of the orthogonalised $U_{IJ}$ tensor

| **Atom** | *X* | *y* | *z* | **U(eq)** |
|---|---|---|---|---|
| O1 | 12894.5(16) | 4.7(12) | 90.2(11) | 20.8(3) |
| O2 | 14365.7(16) | 903.1(13) | 742.4(12) | 22.1(3) |
| O3 | 7441.7(18) | 3892.5(14) | 3979.5(14) | 32.6(4) |
| N1 | 7111.2(19) | 2553.0(14) | 2569.1(14) | 18.0(4) |
| C1 | 12984(2) | 644.7(16) | 640.9(15) | 15.3(4) |
| C2 | 11451(2) | 1103.9(17) | 1166.7(16) | 16.0(4) |
| C3 | 9916(2) | 809.6(17) | 1130.3(16) | 18.1(4) |
| C4 | 8477(2) | 1260.8(17) | 1603.3(16) | 17.7(4) |
| C5 | 8515(2) | 2052.2(17) | 2129.1(16) | 15.6(4) |
| C6 | 10071(2) | 2341.6(17) | 2176.1(17) | 17.9(4) |
| C7 | 11498(2) | 1871.3(17) | 1709.7(16) | 17.4(4) |

| | | | | |
|---|---|---|---|---|
| C8 | 5525(2) | 2156.1(18) | 2709.9(17) | 17.8(4) |
| C9 | 4145(2) | 2858.0(17) | 3198.1(17) | 16.8(4) |
| C10 | 3887(2) | 2464.4(17) | 4424.8(17) | 18.2(4) |
| C11 | 4997(3) | 1491.4(18) | 5245.4(18) | 22.8(4) |
| C12 | 4711(3) | 1136.8(19) | 6420.5(18) | 28.3(5) |
| C13 | 3289(3) | 1704(2) | 6878.0(19) | 30.9(5) |
| C14 | 2207(3) | 2624.1(19) | 6140.1(18) | 26.1(5) |
| C15 | 2468(2) | 3048.4(18) | 4897.3(17) | 19.5(4) |
| C16 | 1369(2) | 3994.2(17) | 4127.1(17) | 19.7(4) |
| C17 | 1626(2) | 4421.2(17) | 2914.9(17) | 18.6(4) |
| C18 | 478(3) | 5381.6(18) | 2129.3(19) | 25.0(5) |
| C19 | 745(3) | 5794.1(19) | 952.1(19) | 28.4(5) |
| C20 | 2201(3) | 5278.3(19) | 473.4(19) | 27.8(5) |
| C21 | 3316(3) | 4353.2(18) | 1184.7(17) | 21.0(4) |
| C22 | 3070(2) | 3860.2(17) | 2436.2(17) | 17.0(4) |
| C23 | 6328(3) | 4399.1(19) | 4442.7(19) | 24.4(5) |
| C24 | 5089(3) | 5445.6(18) | 3724.4(18) | 23.8(5) |
| C25 | 5194(3) | 5870(2) | 2509(2) | 33.0(5) |
| C26 | 4040(3) | 6838(2) | 1835(2) | 39.4(6) |
| C27 | 2770(3) | 7389(2) | 2370(2) | 41.2(6) |
| C28 | 2658(3) | 6988(2) | 3570(2) | 33.0(5) |
| C29 | 3811(3) | 6014.5(18) | 4264.5(19) | 24.6(5) |

**Table S4.** Anisotropic Displacement Parameters (Å$^2$×10$^3$) for L3. The Anisotropic displacement factor exponent takes the form: $-2\pi^2[h^2a^{*2}U_{11}+2hka^*b^*U_{12}+\ldots]$

| **Atom** | **$U_{11}$** | **$U_{22}$** | **$U_{33}$** | **$U_{12}$** | **$U_{13}$** | **$U_{23}$** |
|---|---|---|---|---|---|---|
| O1 | 18.6(7) | 25.0(8) | 24.6(7) | -2.2(6) | 1.3(6) | -17.1(6) |
| O2 | 12.4(7) | 32.1(9) | 26.3(8) | -4.1(6) | 0.6(6) | -16.5(7) |
| O3 | 22.5(8) | 43.1(10) | 49.1(10) | -9.1(7) | 6.0(7) | -35.5(8) |
| N1 | 12.5(8) | 20.6(9) | 27.6(9) | -3.7(7) | 1.8(7) | -16.5(7) |
| C1 | 15.1(9) | 15.3(10) | 13.0(9) | -0.7(7) | -2.3(7) | -3.9(8) |
| C2 | 14.7(9) | 17.1(10) | 14.8(9) | -1.6(8) | -0.5(7) | -6.0(8) |
| C3 | 19.2(10) | 20.0(11) | 19.6(10) | -2.9(8) | -1.9(8) | -12.4(8) |
| C4 | 13.2(9) | 21.3(11) | 21.8(10) | -4.4(8) | -1.6(8) | -10.9(8) |
| C5 | 15.0(9) | 16.3(10) | 13.7(9) | -0.1(7) | -2.9(7) | -5.1(8) |
| C6 | 15.8(10) | 19.5(10) | 23.9(10) | -2.8(8) | -2.4(8) | -13.9(8) |
| C7 | 13.2(9) | 20.3(11) | 21.2(10) | -3.8(8) | -2.2(8) | -10.1(8) |
| C8 | 13.1(9) | 21.0(11) | 21.9(10) | -3.1(8) | -0.7(8) | -11.1(8) |
| C9 | 13.4(9) | 18.5(10) | 23.4(10) | -5.9(8) | 0.7(8) | -12.3(8) |
| C10 | 14.7(9) | 19.4(11) | 24.9(10) | -7.1(8) | 0.1(8) | -11.5(8) |

| | | | | | | |
|---|---|---|---|---|---|---|
| C11 | 21.3(11) | 20.5(11) | 27.4(11) | -6.0(8) | -3.5(9) | -8.9(9) |
| C12 | 34.6(12) | 23.2(12) | 26.3(11) | -10.5(10) | -10.1(10) | -4.6(9) |
| C13 | 45.6(14) | 33.3(13) | 19.3(11) | -20.3(11) | 0.4(10) | -10.3(10) |
| C14 | 29.1(12) | 29.9(12) | 25.7(11) | -12.3(10) | 7.0(9) | -16.4(10) |
| C15 | 19.4(10) | 21.0(11) | 23.1(10) | -8.9(8) | 2.9(8) | -12.3(9) |
| C16 | 14.0(10) | 22.8(11) | 29.4(11) | -5.8(8) | 4.6(8) | -17.7(9) |
| C17 | 15.9(9) | 16.0(10) | 27.2(11) | -5.0(8) | -0.2(8) | -11.0(8) |
| C18 | 17.8(10) | 21.4(11) | 37.4(12) | 1.4(8) | -3.6(9) | -15.2(10) |
| C19 | 27.5(12) | 20.5(11) | 33.3(12) | 1.1(9) | -11.9(10) | -7.2(10) |
| C20 | 35.0(12) | 25.2(12) | 21.4(11) | -8.7(10) | -4.1(9) | -5.5(9) |
| C21 | 22.2(10) | 21.7(11) | 22.0(10) | -8.6(8) | 2.6(8) | -10.3(9) |
| C22 | 14.1(9) | 17.2(10) | 23.4(10) | -5.9(8) | -0.4(8) | -10.4(8) |
| C23 | 21.1(11) | 27.7(12) | 35.5(12) | -13.2(9) | 3.5(9) | -20.8(10) |
| C24 | 26.2(11) | 24.6(12) | 27.4(11) | -14.2(9) | 0.6(9) | -13.0(9) |
| C25 | 40.8(14) | 36.9(14) | 31.0(12) | -23.1(11) | 6.2(10) | -17.8(11) |
| C26 | 59.5(17) | 34.6(14) | 26.6(12) | -27.4(13) | -6.4(12) | -4.9(11) |
| C27 | 50.9(16) | 24.9(13) | 45.9(15) | -13.9(11) | -18.2(13) | -4.9(11) |
| C28 | 32.1(13) | 23.3(12) | 47.3(14) | -7.6(10) | -6.4(11) | -15.4(11) |
| C29 | 24.6(11) | 23.0(11) | 32.3(12) | -10.5(9) | -2.2(9) | -13.9(9) |

**Table S5.** Hydrogen Atom Coordinates (Å×$10^4$) and Isotropic Displacement Parameters (Å$^2$×$10^3$) for L3

| **Atom** | *X* | *y* | *z* | **U(eq)** |
|---|---|---|---|---|
| H1 | 7161.9(19) | 3121.5(14) | 2770.4(14) | 21.6(4) |
| H3 | 9869(2) | 297.2(17) | 778.4(16) | 21.7(5) |
| H4 | 7470(2) | 1044.4(17) | 1577.6(16) | 21.3(5) |
| H6 | 10127(2) | 2854.4(17) | 2525.8(17) | 21.4(5) |
| H7 | 12515(2) | 2063.9(17) | 1753.6(16) | 20.9(5) |
| H8a | 5214(2) | 2247.9(18) | 1953.9(17) | 21.4(5) |
| H8b | 5655(2) | 1309.3(18) | 3234.1(17) | 21.4(5) |
| H11 | 5928(3) | 1097.3(18) | 4965.6(18) | 27.3(5) |
| H12 | 5458(3) | 512.9(19) | 6933.3(18) | 33.9(6) |
| H13 | 3100(3) | 1444(2) | 7686.2(19) | 37.1(6) |
| H14 | 1274(3) | 2986.8(19) | 6449.6(18) | 31.3(6) |
| H16 | 429(2) | 4353.3(17) | 4433.9(17) | 23.7(5) |
| H18 | -474(3) | 5730.5(18) | 2434.8(19) | 30.0(6) |
| H19 | -24(3) | 6415.1(19) | 456.3(19) | 34.0(6) |
| H20 | 2394(3) | 5577.7(19) | -336.8(19) | 33.4(6) |
| H21 | 4263(3) | 4031.6(18) | 850.7(17) | 25.3(5) |
| H25 | 6045(3) | 5502(2) | 2146(2) | 39.5(7) |
| H26 | 4118(3) | 7119(2) | 1021(2) | 47.3(7) |
| H27 | 1989(3) | 8034(2) | 1914(2) | 49.4(8) |
| H28 | 1807(3) | 7367(2) | 3922(2) | 39.6(6) |
| H2 | 15270(40) | 570(40) | 400(30) | 131(16) |

**Table S6.** Multilevel analysis of the molecular structure of L3

| № | Node degrees | Ωi, % | Dimensionality of net | Topological type |
|---|---|---|---|---|
| 1 | 10,16-c | 1.11 | 3D | New topology with Point Symbol: $\{3^{24}.4^{19}.5^{2}\}\{3^{42}.4^{61}.5^{17}\}_2$ |
| 2 | 10,13-c | 2.04 | 3D | 10,13T91 |
| 3 | 7-c | 7.38 | 3D | bct/I 4/m m m->C 2/c (-a+b-2c,a+b,a-b; 1/4,1/4,1/4);Bond sets: 1,3,4,6,7,8:bct |
| 4 | 6-c | 7.52 | 3D | msw |
| 5 | 5-c | 8.05 | 3D | bnn |
| 6 | 3-c | 8.99 | 2D | hcb |
| 7 | 2-c | 12.87 | 1D | 2C1 |
| 8 | 12-c | 15.16 | 0D | 1M2-1 |

**Table S7.** Optimized geometrical parameters of L3

| Parameter Bond length (Å) | B3LYP/6-311++G(d,p) | Experimental | Parameter Bond angle (º) | B3LYP/6-311++G(d,p) | Experimental |
|---|---|---|---|---|---|
| O1-C5 | 1.2214 | 1.27 | C5-O2-H42 | 105.8913 | 111.48 |
| O2-C5 | 1.3672 | 1.28 | H4-N3-C11 | 117.5461 | 119.08 |
| O2-H42 | 0.9709 | 1.24 | H4-N3-C16 | 117.691 | 119.10 |
| N3-H4 | 1.0135 | 0.88 | C11-N3-C16 | 123.559 | 121.81 |
| N3-C11 | 1.3711 | 1.36 | N3-H4-O43 | 169.9926 | 155.39 |
| N3-C16 | 1.457 | 1.45 | O1-C5-O2 | 120.7873 | 122.09 |
| H4-O43 | 2.0903 | 2.15 | O1-C5-C6 | 125.7466 | 118.91 |
| C5-C6 | 1.4707 | 1.46 | O2-C5-C6 | 113.4661 | 118.98 |
| C6-C7 | 1.4033 | 1.39 | C5-C6-C7 | 118.8126 | 121.44 |
| C6-C14 | 1.4102 | 1.40 | C5-C6-C14 | 122.7316 | 120.05 |
| C7-H8 | 1.0853 | 0.95 | C7-C6-C14 | 118.4558 | 118.49 |

| | | | | | |
|---|---|---|---|---|---|
| C7-C9 | 1.3892 | 1.37 | C6-C7-H8 | 118.5712 | 119.40 |
| C9-H10 | 1.0841 | 0.95 | C6-C7-C9 | 121.2813 | 121.29 |
| C9-C11 | 1.4159 | 1.41 | H8-C7-C9 | 120.1474 | 119.35 |
| C11-C12 | 1.4188 | 1.41 | C7-C9-H10 | 119.2326 | 119.82 |
| C12-H13 | 1.086 | 0.94 | C7-C9-C11 | 120.3699 | 120.35 |
| C12-C14 | 1.3836 | 1.37 | H10-C9-C11 | 120.3974 | 119.82 |
| C14-H15 | 1.0844 | 0.95 | N3-C11-C9 | 122.2322 | 122.23 |
| O43-C44 | 1.232 | 1.22 | N3-C11-C12 | 119.5457 | 119.35 |
| C44-C45 | 1.4888 | 1.48 | C9-C11-C12 | 118.2186 | 118.41 |
| C44-C58 | 1.4898 | 1.48 | C11-C12-H13 | 118.8463 | 119.83 |
| C45-C46 | 1.4112 | 1.40 | C11-C12-C14 | 120.7838 | 120.34 |
| C45-C53 | 1.4014 | 1.38 | H13-C12-C14 | 120.3697 | 119.82 |
| C46-C47 | 1.4002 | 1.39 | C6-C14-C12 | 120.8903 | 121.14 |
| C46-C56 | 1.4923 | 1.48 | C6-C14-H15 | 119.4509 | 119.43 |
| C47-H48 | 1.0848 | 0.95 | C12-C14-H15 | 119.6588 | 119.42 |
| C47-C49 | 1.3941 | 1.37 | O43-C44-C45 | 121.2551 | 121.01 |
| | | | O43-C44-C58 | 120.7711 | 120.64 |
| | | | C45-C44-C58 | 117.9734 | 118.33 |
| | | | C44-C45-C46 | 120.9789 | 120.56 |
| | | | C44-C45-C53 | 119.3399 | 119.98 |
| | | | C45-C46-C56 | 121.227 | 121.10 |

| | | | | | |
|---|---|---|---|---|---|
| | | | C47-C46-C56 | 119.0481 | 119.50 |
| | | | C46-C47-H48 | 118.4252 | 119.75 |

**Table S8.** Calculated vibrational frequencies ($cm^{-1}$) assignments of L3 based on B3LYP/6–311++G(d,p) basis set

| Mode No | Experimental wave number ($cm^{-1}$) | Theoretical wave number ($cm^{-1}$) | | $I_{IR}$[c] | $I_{RAMAN}$[d] | Assignments (PED)[(a,b)] |
|---|---|---|---|---|---|---|
| | FT-IR | Unscaled | Scaled | | | |
| 192 | 3351 | 3631.52 | 3489.891 | 50.87 | 9.018704 | γOH |
| 191 | 3290 | 3572.24 | 3432.923 | 564.05 | 100 | γNH |
| 190 | 3110 | 3247.43 | 3120.78 | 4.78 | 0.847443 | γCH |
| 189 | - | 3242.08 | 3115.639 | 5.95 | 1.054871 | γCH |
| 188 | - | 3242.01 | 3115.572 | 10.22 | 1.811896 | γCH |
| 187 | - | 3238.82 | 3112.506 | 7.12 | 1.262299 | γCH |
| 186 | - | 3237.71 | 3111.439 | 0.9294 | 0.164773 | γCH |
| 185 | - | 3237.30 | 3111.045 | 10.63 | 1.884585 | γCH |
| 184 | - | 3236.30 | 3110.084 | 25.77 | 4.568744 | γCH |
| 183 | - | 3234.68 | 3108.527 | 16.91 | 2.997961 | γCH |
| 182 | - | 3224.94 | 3099.167 | 9.04 | 1.602695 | γCH |
| 181 | - | 3222.75 | 3097.063 | 12.02 | 2.131017 | γCH |
| 180 | - | 3219.91 | 3094.334 | 34.37 | 6.093431 | γCH |
| 179 | - | 3218.73 | 3093.201 | 34.51 | 6.118252 | γCH |
| 178 | - | 3217.10 | 3091.633 | 8.33 | 1.476819 | γCH |
| 177 | - | 3214.28 | 3088.923 | 5.75 | 1.019413 | γCH |
| 176 | - | 3208.11 | 3082.994 | 5.25 | 0.930769 | γCH |
| 175 | - | 3207.46 | 3082.369 | 4.20 | 0.744615 | γCH |

| | | | | | | |
|---|---|---|---|---|---|---|
| 174 | - | 3202.04 | 3077.16 | 23.08 | 4.091836 | γCH |
| 173 | - | 3202.04 | 3077.16 | 2.74 | 0.485773 | γCH |
| 172 | - | 3190.36 | 3065.936 | 6.49 | 1.150607 | γCH |
| 171 | - | 3187.88 | 3063.553 | 3.36 | 0.595692 | γCH |
| 170 | - | 3184.30 | 3060.112 | 2.18 | 0.386491 | γCH |
| 169 | - | 3044.66 | 2925.918 | 21.65 | 3.838312 | γCH |
| 168 | 2852 | 3025.86 | 2907.851 | 45.54 | 8.073752 | γCH |
| 167 | 1750 | 1717.67 | 1650.681 | 309.36 | 54.8462 | γCO+ γCC+ βHOC+ βHCC |
| 166 | - | 1687.94 | 1622.11 | 10.56 | 1.872174 | γCC+ βHCC |
| 165 | 1601 | 1683.56 | 1617.901 | 53.44 | 9.474337 | γCC+ γCO+ βHCC |
| 164 | | 1680.11 | 1614.586 | 1.46 | 0.258842 | γCC+ βHCC |
| 163 | - | 1672.31 | 1607.09 | 541.02 | 95.91703 | γCO+ γCC+ βHCH+ βHCO+ βHCC |
| 162 | - | 1667.26 | 1602.237 | 175.49 | 31.11249 | γCO+ γCC+ βHCC |
| 161 | - | 1649.31 | 1584.987 | 2.25 | 0.398901 | γCC+ βHCC |
| 160 | 1550 | 1637.84 | 1573.964 | 1.12 | 0.198564 | γCC+ βHCC |
| 159 | - | 1636.85 | 1573.013 | 95.57 | 16.94353 | γCC+ βHCN+ βHCC+ βHCH |
| 158 | - | 1626.13 | 1562.711 | 345.36 | 61.22861 | γCO+ γCC+ βHCC |
| 157 | - | 1623.92 | 1560.587 | 2.13 | 0.377626 | γCO+ γCC+ βHCC |
| 156 | - | 1612.13 | 1549.257 | 21.90 | 3.882635 | γCO+ γCC+ βHCC+ βHCH |
| 155 | - | 1611.74 | 1548.882 | 53.13 | 9.419378 | γCO+ γCC+ βHCC+ βHCH |
| 154 | - | 1600.80 | 1538.369 | 3.26 | 0.577963 | γCC+ γCN+ βHCC+ βHCH |
| 153 | - | 1585.68 | 1523.838 | 217.61 | 38.57991 | γCC+ γCN+ βHCC+ βHCH+ βOCO |

| | | | | | | |
|---|---|---|---|---|---|---|
| 152 | 1512 | 1584.27 | 1522.483 | 30.41 | 5.391366 | γCC+ γCN+ βHCC+ βHCH+ βOCO |
| 151 | 1450 | 1556.74 | 1496.027 | 13.07 | 2.31717 | γCC +βCNH+ βHCC+ βHCH |
| 143 | 1423 | 1484.60 | 1426.701 | 38.24 | 6.779541 | γCC+ βCNH+ βHCC+ βHCH |

**Table S9.** The values of calculated dipole moment μ(D), polarizability (α0), first order hyperpolarizability, (βtot) components of **L3**

| **Parameters** | **B3LYP/6-311++G(d,p)** | **Parameters** | **B3LYP/6-311++G(d,p)** |
|---|---|---|---|
| $\mu_x$ | 6.5518 | $\beta_{xxx}$ | 341.9681 |
| $\mu_y$ | 0.0381 | $\beta_{yxx}$ | 21.1670 |
| $\mu_z$ | 0.5198 | $\beta_{xyy}$ | -97.2962 |
| **μ(D)** | **6.5725** | $\beta_{yyy}$ | -123.1739 |
| $\alpha_{xx}$ | -268.8332 | $\beta_{zxx}$ | -36.0540 |
| $\alpha_{xy}$ | 22.9133 | $\beta_{xyz}$ | -22.5801 |
| $\alpha_{yy}$ | -219.3266 | $\beta_{zyy}$ | 22.8157 |
| $\alpha_{xz}$ | -6.3929 | $\beta_{xzz}$ | 16.9878 |
| $\alpha_{yz}$ | -10.9098 | $\beta_{yzz}$ | 9.2168 |
| $\alpha_{zz}$ | -218.8156 | $\beta_{zzz}$ | 10.1868 |
| **$\alpha_0$ (e.s.u)** | **$3.4\times10^{-23}$** | **$\beta_{tot}$ (e.s.u)** | **$2.398\times10^{-30}$** |

**Table S10.** Calculated energy values of L3 by B3LYP/6– 311++G(d,p) method

| **Parameter** | **Values** |
|---|---|
| $E_{Homo}$(eV) | -5.51 |
| $E_{Lumo}$(eV) | -1.86 |
| Ionization potential | 5.51 |
| Electron affinity | 1.86 |
| Energy gap(eV) | 3.64 |
| Electronegativity | 3.68 |
| Chemical potential | -3.68 |
| Chemical hardness | 1.825 |
| Chemical softness | 0.547 |
| Electrophilicity index | 3.71 |

**Table S11.** Surface property information in Hirshfeld for **L3**

| **Mode** | **Minimum interaction** | **Mean interaction** | **Maximum interaction** |
|---|---|---|---|
| **$D_{norm}$** | -0.787 | 0.599 | 2.364 |
| **$D_i$** | 0.563 | 1.774 | 3.620 |
| **$D_e$** | 0.563 | 1.799 | 3.694 |
| **Shape Index** | -0.995 | 0.209 | 0.998 |
| **Curvedness** | -3.625 | -0.997 | 0.397 |

| | | | |
|---|---|---|---|
| **Fragment patches** | 0.0 | 9.5084 | 18.0000 |

**Table S12.** 2-D finger print percentage of the total surface area for closed contact between atoms inside and outside the surface for **L3**

| **Atoms Surface %** | **O** | **N** | **H** | **C** |
|---|---|---|---|---|
| **O** | - | - | 17.6 | 0.4 |
| **N** | - | - | 0.4 | - |
| **H** | 17.6 | 0.4 | 47.4 | 29.4 |
| **C** | 0.4 | - | 29.4 | 4.9 |

**Table S13**. Hydrogen bonding and molecular docking with centromere associated protein inhibitor protein targets

| **S. No.** | **Protein (PDB ID)** | **No of residue** | **Bond distance (Å)** | **Inhibition Constant (micromolar)** | **Binding Energy (kcal/mol)** | **Refrence RMSD (Å)** |
|---|---|---|---|---|---|---|
| 1 | 5E1S | 3 | 2.34 | 0.012 | -10.8 | 6.396 |

**References**


1. International Tables for X-Ray Crystallography, Vol. III, Kynoch Press, Birmingham, England, 1952.
2. O. V. Dolomanov, L. J. Bourhis, R. J. Gildea, J. A. K. Howard and H. Puschmann, *J. Appl. Crystallogr.*, 42 (2009) 339–341.
3. Blatov V. A., Shevchenko A. P., Proserpio D. M. Applied topological analysis of crystal structures with the program package ToposPro, Cryst. Growth Des., 2014, 14, 3576–3586. doi: 10.1021/cg500498k
4. O'Keeffe M., Peskov M. A., Ramsden S. J., Yaghi O. M. The reticular chemistry structure resource (RCSR) database of, and symbols for, crystal nets. Acc. Chem. Res., 2008, 41, 1782-1789. doi: 10.1021/ar800124u
5. Alexandrov E. V., Blatov V. A., Kochetkov A. V., Proserpio D.M. Underlying nets in three-periodic coordination polymers: topology, taxonomy and prediction from a computer-aided analysis of the Cambridge Structural Database. CrystEngComm 2011, 13, 3947-3958. doi: 10.1039/C0CE00636J
6. Blatov V. A., Shevchenko A. P. Simplify to understand: how to elucidate crystal structures? Struct. Chem. 2021, doi: 10.1007/s11224-020-01724-4
7. Alexandrov E. V., Shevchenko A. P., Blatov V. A. Topological Databases: Why Do We Need Them for Design of Coordination Polymers? Cryst. Growth Des. 2019, 19, 2604-2614. doi: 10.1021/acs.cgd.8b01721.
8. G.A. Petersson, M.A. Al-Laham, *J. Chem. Phys.* **1991**, 94 (9), 6081.
9. G.A. Petersson, A. Bennet, T.G. Tensfeld, M.A. Al-Laham, W.A. Shirley, J. Mantzaris, *J. Chem. Phys.* **1988**, 89(4), 2193.
10. M.J. Frisch, et al., Gaussian 09, Revision E.01, Gaussian, Inc., Wallingford CT, 2009.
11. F. Neese, *Mol. Sci.* **2012**, 2, 73.
12. T. Lu. F. Chen, *J. Comp. Chem.* **2012**,33, 580.
13. A. Daina, O. Michielin, V. Zoete, *Scientific Reports*, **2017**, 7, 42717.
14. Origin 8.0, OriginLab Corp., Northampton, MA.
15. F.L. Hirshfeld, *Theor. Chim.Acta.***1977**,44, 129–138.

16. M. A. Spackman, D. Jayatilaka, *Cryst. Eng. Comm.***2009**,11, 19–32.
17. W. Wang, Y. Ling, L. J. Yang, Q. L. Liu, Y. H. Luo, B. W. Sun, *Res. Chem. Intermed*. **2016**,42 (4), 3157–3168.
18. D.A. Safin, K. Robeyns, Y. Garcia, *Cryst. Eng. Comm*. **2016**,18, 7284- 7296.
19. N.P.G. Roeges, A Guide to the Complete Interpretation of the Infrared Spectra of Organic Structures, Wiley, New York, 1994. (b) N. Rekik, N. Issaoui, H. Ghalla, B. Oujia, M.J. Wojcik, Infrared spectral density of H-bonds within the strong anharmonic coupling theory: Indirect relaxation effect, Journal of Molecular Structure 844–845 (2007) 21–31.
20. P.S.Ganeshvar, M.Kanagaraj, S.Gunasekaran, T.Gnanasambandan. International J. of scientific & Engineering Research, Vol 7 (2016) ISSN 2229-5518.
